# Genetic analysis of epistasis between nucleotide excision repair and homologous recombination in the recovery of persisters after fluoroquinolone treatment

**DOI:** 10.1371/journal.pgen.1012305

**Published:** 2026-09-21

**Authors:** Nashaly Soto-Echevarria, Annabel S. Lemma, Mark P. Brynildsen

**Affiliations:** 1 Department of Molecular Biology, Princeton University, Princeton, New Jersey, United States of America; 2 Department of Chemical and Biological Engineering, Princeton University, Princeton, New Jersey, United States of America; 3 Omenn-Darling Bioengineering Institute, Princeton University, Princeton, New Jersey, United States of America; Norwegian University of Life Sciences: Norges miljo- og biovitenskapelige universitet, NORWAY

## Abstract

Stationary-phase cultures contain high abundances of persisters, which are bacterial cells that are hyper-tolerant to antibiotics due to phenotypic reasons. Fluoroquinolones (FQs) are one of the most effective antibiotic classes for the treatment of non-growing bacteria, such as those found in stationary-phase cultures, and persisters in those populations have been found to survive FQ treatment by repairing the DNA damage caused by the antibiotic rather than by avoiding damage through inactivity. Interestingly, previous work demonstrated that transient growth inhibition after the conclusion of FQ treatment significantly increased persister levels from stationary-phase *Escherichia coli* populations if *recA*, a mediator of homologous recombination (HR), or *uvrD*, a component of nucleotide excision repair (NER), were present. Here, we sought to identify additional epistatic interaction partners in that persister recovery network by deleting DNA repair enzymes known to interact with RecA or UvrD. We found that *uvrA*, *uvrB*, and *mfd* also epistatically interact with *recA* in FQ persister recovery, whereas *recB* and *recC* are additional epistatic interaction partners of *uvrD*. While these results indicated that HR and NER can contribute to persister recovery, different combinations of genetic mutants suggested that the phenomenon is more nuanced than compensation for loss of one repair pathway by another. Specifically, loss of *recA* had farther reaching epistatic consequences than loss of other HR genes, and loss of *uvrD* had a greater impact on the network than any other NER gene. Delving deeper into the roles of RecA and UvrD, we found that the recombination function of RecA, via RecA(N304D), and helicase function of UvrD, via UvrD(R284A), were required for their participation in FQ persister recovery. Collectively, the data presented here deepen understanding of the roles of HR and NER machinery in the recovery FQ persisters and further emphasize the important roles of RecA and UvrD in this phenomenon.

## Author summary

Persisters are rare phenotypic variants that survive antibiotic treatments that kill the vast majority of cells in cultures, and evidence has accumulated to support their role in chronic and recurrent infections. Persisters are abundant in populations where nutrients have been exhausted, such as stationary-phase cultures, and FQs are one of the few antibiotic classes that remain efficacious against such populations. Previous work found that transient growth inhibition after FQ treatment increased the abundance of persisters from stationary-phase *E. coli* populations by providing time for bacteria to repair FQ-induced DNA damage before resuming growth. Further, an epistasis between *recA* (a mediator of homologous recombination) and *uvrD* (a participant in nucleotide excision repair) was uncovered in the phenomenon. Here we adopted a genetic approach to identify additional members of this epistatic network by focusing on known interaction partners of RecA and UvrD. We found that *uvrA*, *uvrB*, and *mfd* also epistatically interact with *recA*, whereas *recB* and *recC* are additional epistatic interaction partners of *uvrD*. Additional analyses revealed the central roles of *recA* and *uvrD* in this epistatic network that has the capacity to reduce the efficacy of FQs by up to an order of magnitude.

## 1. Introduction

Persisters survive antibiotics by occupying transient phenotypic states that die more slowly during antibiotic treatments than the majority of their clonal neighbors [1–3]. After antibiotic exposure concludes, persisters exit those tolerant phenotypic states and give rise to new populations that are as susceptible to antibiotics as the original cultures [1–4]. Persisters have been detected in a wide variety of bacterial species, are thought to contribute to infection relapse, and have been shown to facilitate the development of antibiotic-resistant mutants [4–11]. Overall, increased understanding of persister survival tactics has the potential to improve outcomes for relapsing infections [7,12].

Fluoroquinolones (FQs) are antibiotics that target type II topoisomerases (e.g., DNA gyrase, topoisomerase IV) that depending on the concentration used produce single and/or double strand breaks (SSBs and DSBs) in DNA [13–17]. Previous work has shown that persisters survive FQ treatment by repairing DNA damage that ensued from topoisomerase corruption, rather than by avoiding FQ-induced DNA damage through topoisomerase inactivity [18–21]. Two prominent DNA repair pathways used by FQ persisters are homologous recombination (HR) and nucleotide excision repair (NER) [10,19,21–26]. HR can repair DSBs via the RecBCD pathway and single strand lesions via the RecFOR pathway [27–29]. RecA is a key HR enzyme as both pathways load RecA onto nascent single-stranded DNA (ssDNA); for DSBs, RecBCD will help load RecA onto ssDNA generated after Chi site recognition [30], whereas for single strand gaps (SSGs), RecFOR binds to double-stranded DNA (dsDNA)-ssDNA junctions and facilitates RecA loading in a 5’ to 3’ direction [31]. Once loaded, ssDNA-bound RecA forms helical filaments that protect damaged DNA from further degradation, initiate the SOS response, and facilitate strand exchange [27–29]. NER can be divided into three sub-pathways which sense and fix DNA damage via the UvrABC machinery [32,33]. In global genomic repair (GGR), DNA damage is sensed through the UvrA_2_B_2_ complex by a change in helix rigidity; in Mfd-mediated transcription coupled repair (TCR), Mfd binds to a stalled RNA polymerase and displaces it by pushing it forward to expose the lesion for UvrA_2_B_2_ binding; and in UvrD-mediated TCR, UvrD binds to a stalled RNA polymerase but backtracks it to expose the damaged site for UvrA_2_B_2_ [32–37]. Once UvrA_2_B_2_ detects the lesion, UvrC is recruited to generate a dual incision 3’ and 5’ from the damaged nucleotides [32,33,35]. In addition to binding stalled RNA polymerase in UvrD-mediated TCR, UvrD plays a key role in all three sub-pathways by releasing UvrC and employing its helicase activity to remove the damaged oligonucleotides [32,33]. Interestingly, our recent work revealed an epistatic interaction between UvrD and RecA in the recovery of persisters from FQ treatment [23], which is the focus of this study.

A growing body of evidence has demonstrated the importance of post-treatment events on FQ persister survival [18,19,23,38]. For example, our group showed that *recA* and the SOS response were not needed by FQ persisters from stationary-phase populations until FQ treatment had ended and cultures were exposed to fresh nutrients [18]. We then found that the relative timing of DNA repair and growth resumption following FQ treatment was a critical parameter in defining persister levels of starving populations [19]. Later, we observed that inhibition of transcription or translation after FQ treatment had ended produced significant increases in persistence from stationary-phase populations [23]. That phenomenon, which we will call inhibition-assisted recovery, required *recA* or *uvrD*, because it was absent from the double deletion mutant, Δ*uvrD* Δ*recA*, but present in the single deletion strains, Δ*recA* and Δ*uvrD* [18]. Notably, the origin of the epistasis was not uncovered, which is a complex task given the large number of interaction partners both RecA and UvrD have [27,28,35,39–42].

In this study, we set out to identify RecA and UvrD interaction partners that participate in the epistasis in inhibition-assisted recovery of *Escherichia coli* FQ persisters. Our results revealed that *uvrA*, *uvrB*, *mfd*, *recB*, and *recC* are all members of the epistatic interaction network along with *recA* and *uvrD*, and that *recA* and *uvrD* exhibit the strongest degrees of epistasis with other genes. Further, analyses of loss of function and impaired function mutants of RecA and UvrD demonstrated that the recombination abilities of RecA and helicase activity of UvrD were critical to this epistatic phenomenon. Overall, the results presented elaborate on an epistasis observed between *recA* and *uvrD* to include additional genes involved in NER and HR DSB repair and provide deeper understanding of the functions of RecA and UvrD that contribute to inhibition-assisted recovery of FQ persisters.

## 2. Results

### 2.1. *uvrA*, *uvrB*, and *mfd* exhibit epistasis with *recA* in the recovery of FQ persisters

**Fig 1:**
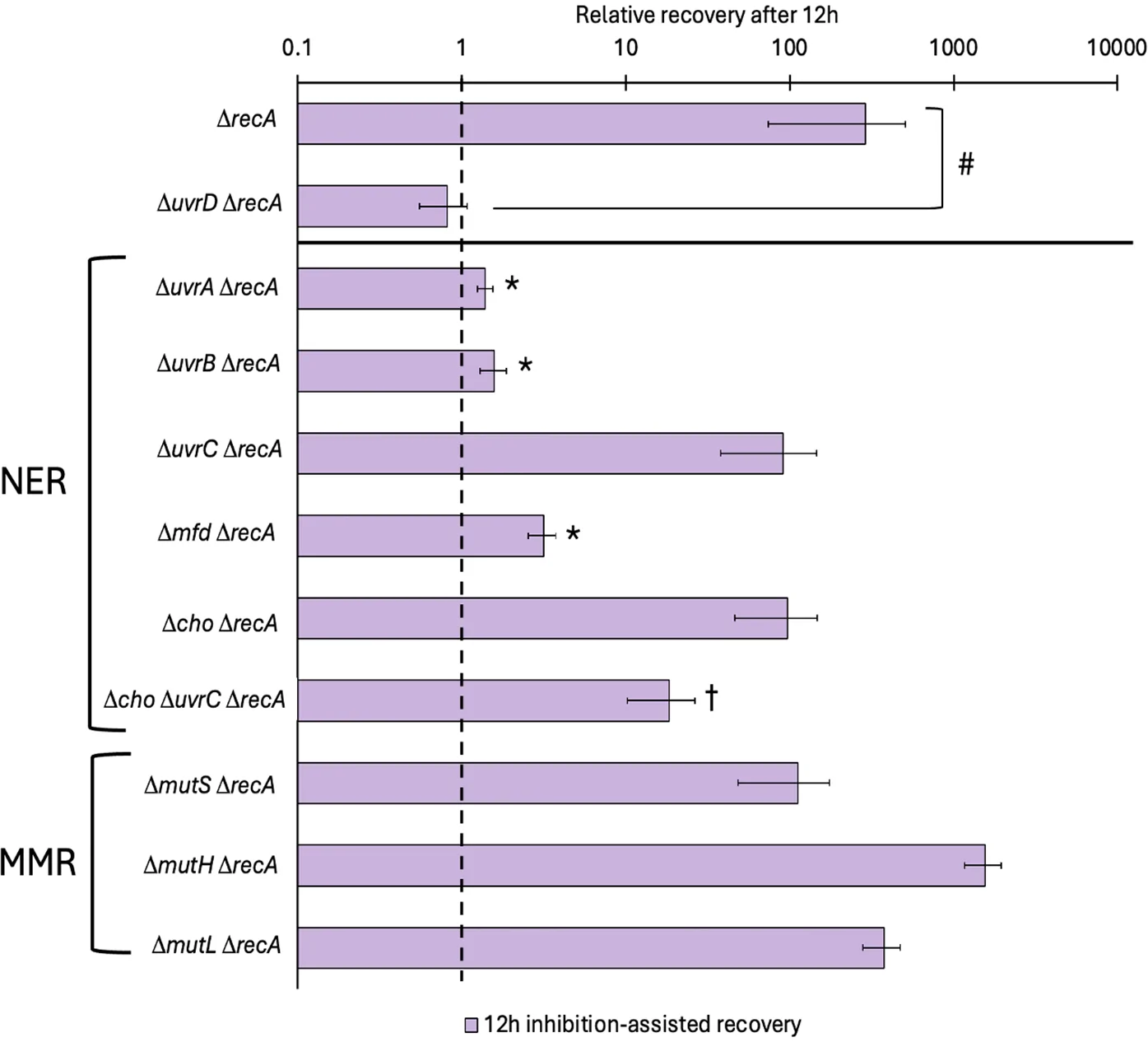
Screen for epistatic interactions with *recA* in FQ persister recovery. Stationary-phase cultures of strains harboring indicated mutations were treated with 5 µg/mL ofloxacin (OFL) and assayed for recovery by washing three times with PBS and plating on LB agar supplemented with 25 µg/mL CAM on top of 0.2 µm filters. After 12 h, filters were transferred to LB agar plates for 24 h. Δ*uvrA* Δ*recA*, Δ*uvrB* Δ*recA*, and Δ*mfd* Δ*recA* strains did not undergo inhibition-assisted recovery, whereas Δ*cho* Δ*uvrC* Δ*recA* exhibited an attenuated response. Strains in this screen contained kanamycin resistance cassettes from the knockout procedure (S1 Table). Data points reflect the mean values of at least three biological replicates with error bars indicating the standard errors of the means. One-way ANOVA with post-hoc Tukey tests were conducted to assess statistical significance. *: reflects statistically significant differences (p ≤ 0.05) between the indicated mutant and Δ*recA*, but not Δ*uvrD* Δ*recA*. †: reflects statistically significant differences (p ≤ 0.05) between the indicated mutant and both Δ*recA* and Δ*uvrD* Δ*recA.* #: reflects statistically significant difference between the indicated references strains.

In previous work, translational inhibition with chloramphenicol (CAM) following FQ treatment increased survival in Δ*recA* and Δ*uvrD*, but not in the Δ*uvrD* Δ*recA* double mutant [23]. We sought to identify additional DNA repair genes that were part of this epistatic network. We began by screening DNA repair genes whose products were known to interact with UvrD for whether their knockouts when combined with Δ*recA* would resemble Δ*uvrD* Δ*recA* in FQ persister recovery assays. We considered genes involved in NER (*uvrA*, *uvrB*, *uvrC*, *mfd*) and mismatch repair (MMR) (*mutS*, *mutH*, *mutL*) due to the prominent role of UvrD in these repair pathways [32,33,39,43,44]. The screen suggested that none of the MMR genes could recapitulate the phenotype of *uvrD* in FQ persister recovery assays, whereas *uvrA*, *uvrB*, and *mfd* did (Fig 1). Specifically, the recoveries of Δ*uvrA* Δ*recA*, Δ*uvrB* Δ*recA*, and Δ*mfd* Δ*recA* resembled that of Δ*uvrD* Δ*recA*, where survival with or without CAM were near equivalent (Fig 1). Interestingly, the phenotype of *uvrC*, the other NER gene that is involved in all three NER pathways (GGR, Mfd-mediated TCR, and UvrD-mediated TCR) [32,33], when knocked out in Δ*recA* did not resemble that of Δ*uvrD* Δ*recA*. *E. coli* encodes another excision nuclease named Cho that interacts with the NER machinery and has homology to UvrC [35]. We considered whether *cho* might be participating in FQ persister recovery and assayed Δ*cho* Δ*recA* and Δ*cho* Δ*uvrC* Δ*recA*. Neither Δ*cho* Δ*recA* nor Δ*cho* Δ*uvrC* Δ*recA* resembled Δ*uvrD* Δ*recA* in FQ persister recovery assays; however, the triple mutant exhibited attenuated recovery (Fig 1).

**Fig 2:**
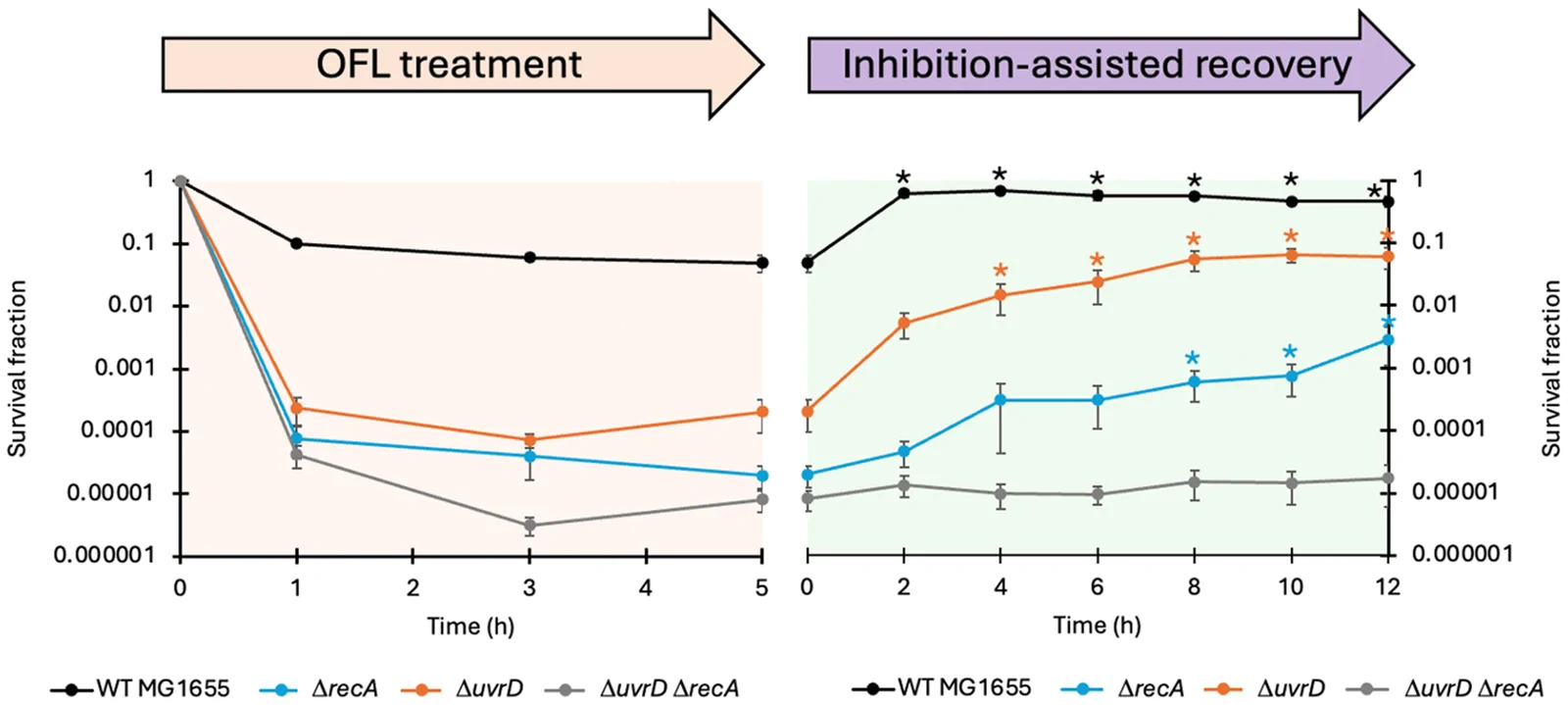
Epistasis between *recA* and *uvrD* in FQ persister recovery occurs with post-treatment translational inhibition. Stationary-phase cells were treated with 5 µg/mL OFL, CFU/mL were monitored, and survival fractions were calculated. At 5 h post-OFL treatment, cells were spotted on filters placed on top of LB agar supplemented with 25 µg/mL CAM. Samples were incubated at 37 °C for the times indicated before being transferred to LB agar without CAM for 24 h. Strains in these assays were cured of kanamycin resistance cassettes (S1 Table). WT, Δ*recA*, and Δ*uvrD* exhibited significant increases in persister levels from post-FQ translational inhibition. However, the recovery period did not increase persistence in Δ*uvrD* Δ*recA*. Data points reflect the mean values of at least three biological replicates with error bars indicating the standard errors of the means. One-way ANOVA with post-hoc Tukey tests were conducted to assess statistical significance. *: indicates statistically significant (p ≤ 0.05) differences between time points and t = 0 h of the same samples.

With these screening results suggesting that loss of *uvrA*, *uvrB,* or *mfd* from Δ*recA* eliminates its ability to undergo inhibition-assisted recovery, we advanced *uvrA*, *uvrB*, and *mfd* to further testing with time-course recovery assays. Importantly, time-course assays reveal the dynamics of recovery, which were different between Δ*recA* and Δ*uvrD* (Fig 2, green-shaded background). For instance, defining the time to recover as a significant increase in persisters that exceeds 10-fold, it can be observed that the time to recover for wild-type (WT) was 2 h, for Δ*uvrD* was 4 h, and for Δ*recA* was 8 h. Such data suggests that the timescale to restore culturability after FQ treatment depends to a greater extent on *recA* than *uvrD* under the conditions considered here.

**Fig 3:**
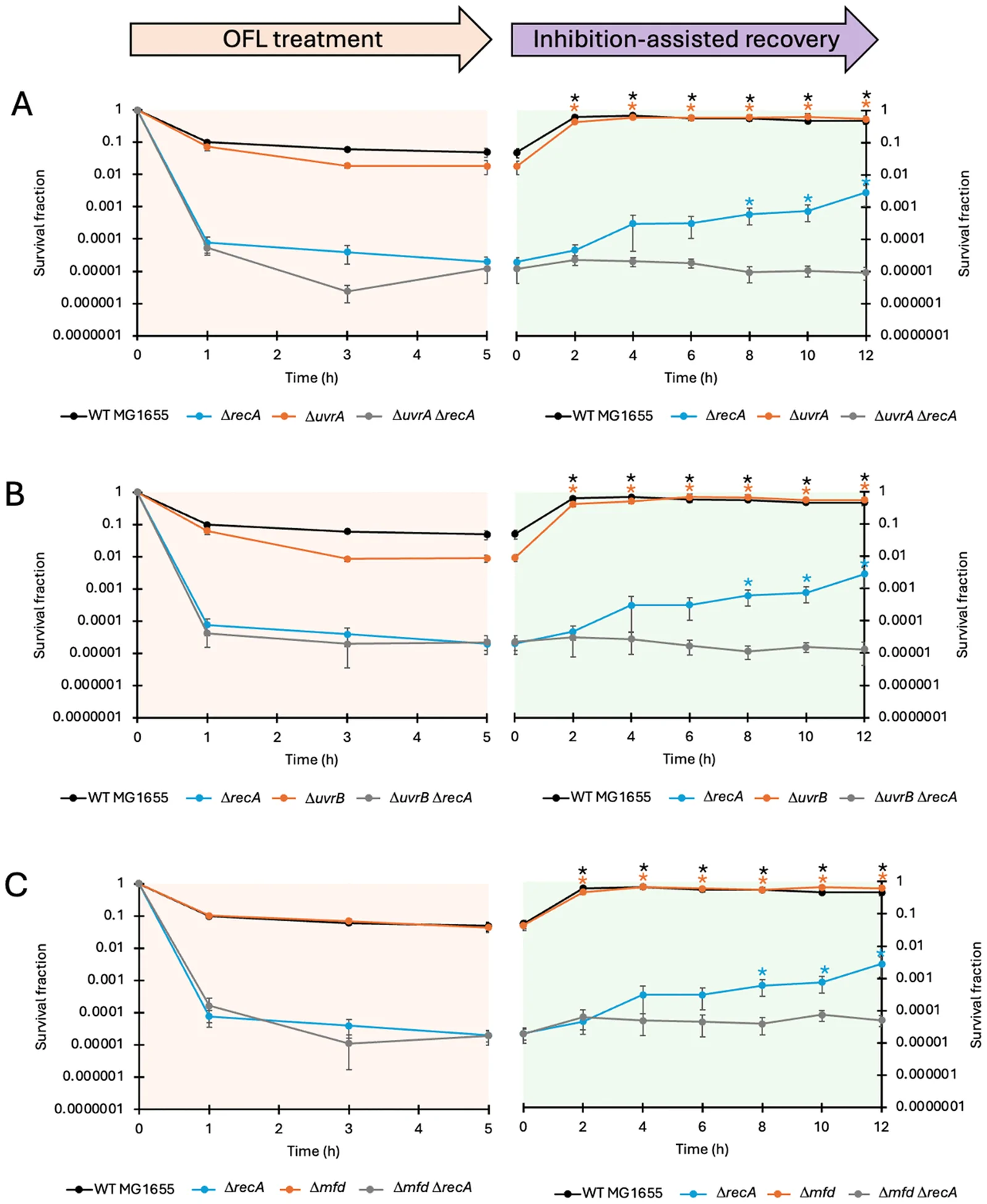
Δ*uvrA*, Δ*uvrB*, and Δ*mfd* recapitulate the phenotype of Δ*uvrD* in FQ persister recovery. Stationary-phase cells were treated with 5 µg/mL OFL, CFU/mL were monitored, and survival fractions were calculated. At 5 h post-OFL treatment, cells were spotted on filters placed on top of LB agar supplemented with 25 µg/mL CAM. Samples were incubated at 37 °C for the times indicated before being transferred to LB agar without CAM for 24 h. Strains in these assays were cured of kanamycin resistance cassettes (S1 Table). Δ*uvrA* **(A)**, Δ*uvrB* **(B)**, Δ*mfd* **(C)**, and Δ*recA* **(A-C)** exhibited significant increases in persister levels from post-FQ translational inhibition. However, the recovery period did not increase persistence in Δ*uvrA* Δ*recA* **(A)**, Δ*uvrB* Δ*recA* **(B)**, or Δ*mfd* Δ*recA* **(C)**. Data points reflect the mean values of at least three biological replicates with error bars indicating the standard errors of the means. One-way ANOVA with post-hoc Tukey tests were conducted to assess statistical significance. *: indicates statistically significant (p ≤ 0.05) differences between time points and t = 0 h of the same samples.

In time-course recovery assays (Fig 3), Δ*uvrA*, Δ*uvrB*, and Δ*mfd* showed significant increases in survival when cultures were exposed to CAM after FQ treatment compared to cultures that were not exposed to CAM (t = 0 h of recovery)*.* That data showed that loss of *uvrA*, *uvrB*, or *mfd* on their own does not eliminate inhibition-assisted recovery. When Δ*uvrA*, Δ*uvrB*, or Δ*mfd* were combined with Δ*recA*, little difference in survival was observed for cultures with or without CAM, which demonstrated that loss of these NER genes in a strain devoid of *recA* eliminates inhibition-assisted recovery (Fig 3). These results recapitulate the phenotype of Δ*uvrD* where in isolation inhibition-assisted recovery of FQ persisters was observed, whereas in combination with Δ*recA* recovery was not seen (Fig 2). Further, inspection of the dynamics revealed recovery that occurred within a 2 h timescale for Δ*uvrA*, Δ*uvrB*, or Δ*mfd*, which resembled the timescales of WT and Δ*uvrD*. In addition, the lack of recovery in all three double deletion strains suggests that *uvrA*, *uvrB*, and *mfd* are involved in processes that enable recovery at times scales of 8 h and later, which is similar to *uvrD*.

To confirm that *uvrA*, *uvrB*, *mfd*, and *uvrD* were components of this epistatic persister recovery network, we performed complementation experiments with the double knockout mutants. Expression of *recA* or the missing NER gene from their native promoters on plasmids restored inhibition-assisted recovery in double knockout mutants, whereas empty vectors had little difference in survival (S1 and S2 Figs). Collectively, these data suggested that *uvrA*, *uvrB*, *mfd,* and *uvrD* exhibit epistasis with *recA* in the phenomenon of inhibition-assisted recovery of FQ persisters.

### 2.2. *recB* and *recC* exhibit epistasis with *uvrD* in the recovery of FQ persisters

**Fig 4:**
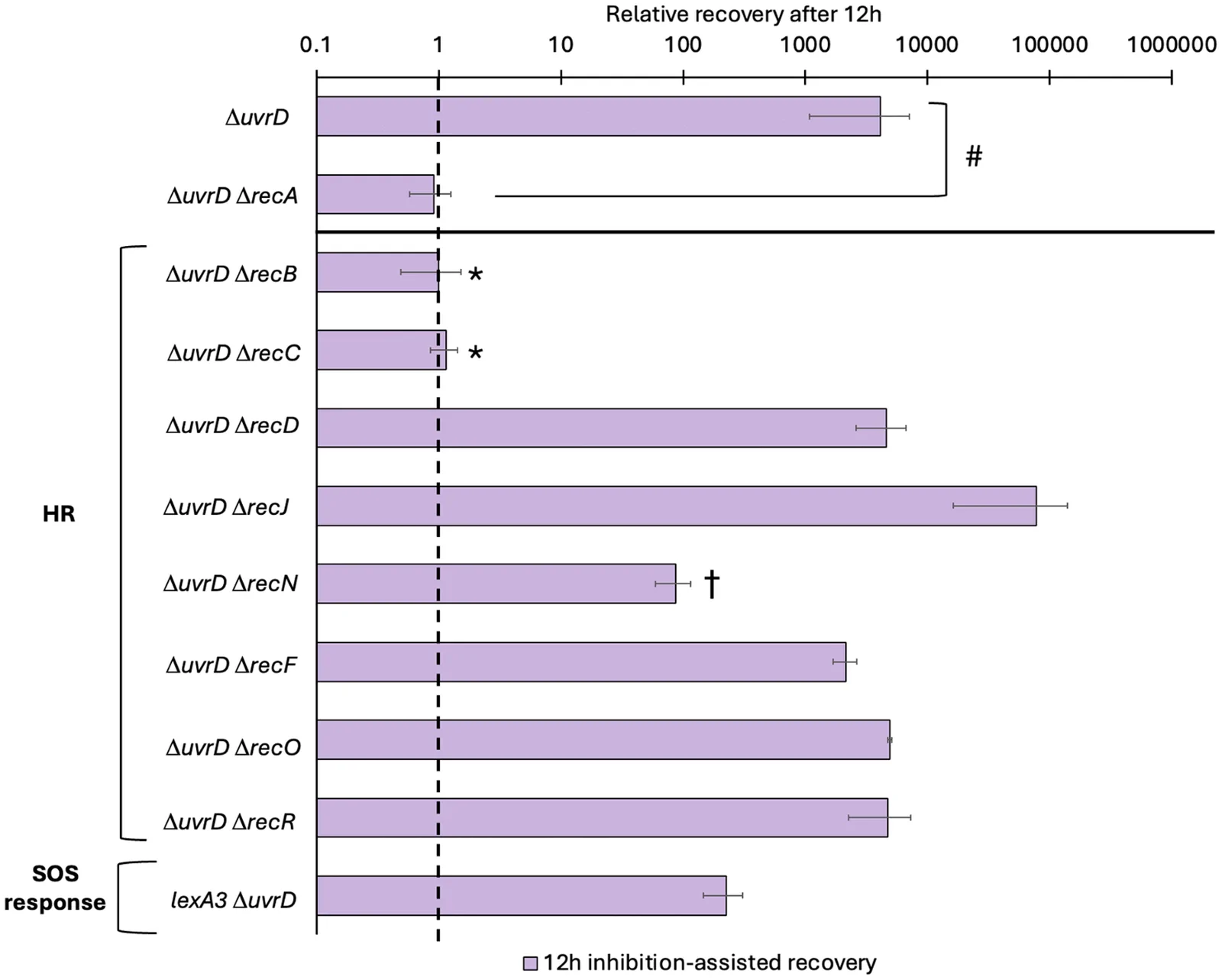
Screen for epistatic interactions with *uvrD* in FQ persister recovery. Stationary-phase cultures of strains harboring indicated mutations were treated with 5 µg/mL OFL and assayed for recovery by washing three times with PBS and plating on LB agar supplemented with 25 µg/mL CAM for 12 h on top of 0.2 µm filters. After 12 h, filters were transferred to LB agar plates for 24 h. Δ*uvrD* Δ*recB* and Δ*uvrD* Δ*recC* did not undergo inhibition-assisted recovery, whereas Δ*uvrD* Δ*recN* exhibited an attenuated response. Strains in this screen contained kanamycin resistance cassettes from the knockout procedures (S1 Table). Data points reflect the mean values of at least three biological replicates with error bars indicating the standard errors of the means. One-way ANOVA with post-hoc Tukey tests were conducted to assess statistical significance. *: reflects statistically significant differences (p ≤ 0.05) between the indicated mutant and Δ*uvrD*, but not Δ*uvrD* Δ*recA*. †: reflects statistically significant differences (p ≤ 0.05) between the indicated mutant and both Δ*uvrD* and Δ*uvrD* Δ*recA.* #: reflects statistically significant difference between the indicated references strains.

To further identify DNA repair genes that are part of the epistatic network responsible for FQ persister recovery, we screened genes whose products were known to interact with RecA for epistasis with *uvrD* [27,45]. Specifically, we screened genes involved in recombinatorial repair (*recB*, *recC*, *recD, recJ, recN, recF, recO, recR*) with knockouts, and the SOS response with an uninducible allele of *lexA* (*lexA3*) [18,27–29,46]. Results suggested that Δ*recD*, Δ*recJ*, Δ*recF*, Δ*recO*, Δ*recR,* and *lexA3* cannot recapitulate the phenotype of Δ*recA* in its epistasis with Δ*uvrD* in FQ persister recovery assays (Fig 4). However, both Δ*uvrD* Δ*recB* and Δ*uvrD* Δ*recC* failed to exhibit inhibition-assisted recovery and thus could recapitulate the phenotype of Δ*uvrD* Δ*recA*, whereas Δ*uvrD* Δ*recN* exhibited attenuated recovery (Fig 4). With these screening results suggesting that loss of *recB* or *recC* from Δ*uvrD* eliminates its ability to undergo inhibition-assisted recovery, we advanced *recB* and *recC* to further testing with time-course recovery assays.

**Fig 5:**
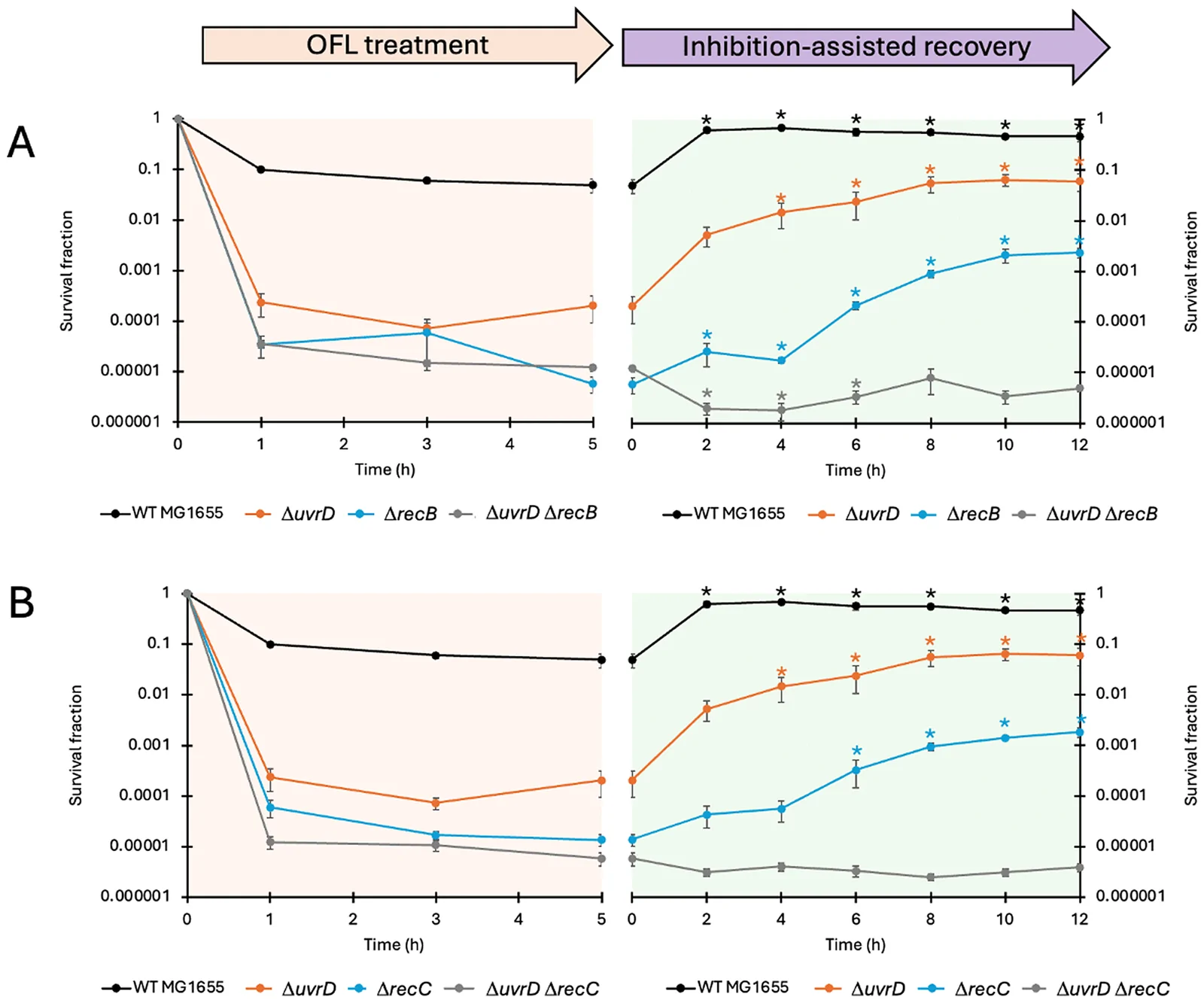
Δ*recB* and Δ*recC* recapitulate the phenotype of Δ*recA* in FQ persister recovery. Stationary-phase cells were treated with 5 µg/mL OFL, CFU/mL were monitored, and survival fractions were calculated. At 5 h post-OFL treatment, cells were spotted on filters placed on top of LB agar supplemented with 25 µg/mL CAM. Samples were incubated at 37 °C for the times indicated before being transferred to LB agar without CAM for 24 h. Strains in these assays were cured of kanamycin resistance cassettes (S1 Table). Δ*recB* **(A)**, Δ*recC* **(B)**, and Δ*uvrD* **(A-B)** exhibited significant increases in persister levels from post-FQ translational inhibition. However, the recovery period did not increase persistence in Δ*uvrD* Δ*recB* **(A)** or Δ*uvrD* Δ*recC* **(B)**. Data points reflect the mean values of at least three biological replicates with error bars indicating the standard errors of the means. One-way ANOVA with post-hoc Tukey tests were conducted to assess statistical significance. *: indicates statistically significant (p ≤ 0.05) differences between time points and t = 0 h of the same samples.

In time course assays, both Δ*recB* and Δ*recC* displayed significant increases in survival when cultures were exposed to CAM after FQ treatment compared to cultures that were not exposed to CAM (Fig 5). That data showed that loss of *recB* or *recC* on their own does not eliminate inhibition-assisted recovery. However, when Δ*recB* or Δ*recC* were combined with Δ*uvrD* little difference in survival was observed for cultures with or without CAM (Fig 5). These results recapitulate the phenotype of Δ*recA* where in isolation inhibition-assisted recovery of FQ persisters was observed, whereas in combination with Δ*uvrD* recovery was not seen (Fig 2). Analysis of the recovery dynamics revealed that the time to recover for Δ*recB* and Δ*recC* was 6 h (Fig 5), which more closely matched the dynamics of Δ*recA* recovery compared to the dynamics of WT, Δ*uvrA*, Δ*uvrB*, Δ*uvrD*, and Δ*mfd*. These results suggest that *recB* and *recC* are involved in processes that enable recovery at timescales of 4 h and earlier, which is similar to *recA.*

To confirm that *recB* and *recC* were components of this epistatic persister recovery network, we performed complementation experiments with the double knockout mutants (S3 Fig). Expression of *recB* and *recC* from their native promoters on plasmids significantly restored inhibition-assisted recovery in double knockout mutants; however, the empty vector pUA66 and expression of *uvrD* from its native promoter on pUA66 presented issues with colony counts falling under the limit of detection (S3 Fig)*.* To address this technical hurdle, we used Tn7-mediated site-specific transposition to insert deleted genes and their native promoters directly into the chromosome (S1 Table) [47]. As an empty control, we incorporated the associated multiple cloning site (MCS) at the same genomic position (S1 Table). Expression of *uvrD* from its native promoter located in the attTn7 site restored inhibition-assisted recovery in both Δ*uvrD* Δ*recB* and Δ*uvrD* Δ*recC*, whereas the empty control had little difference in survival from before and after 12 h of recovery in Δ*uvrD* Δ*recB* (S3 Fig). For Δ*uvrD* Δ*recC*, the empty control still resulted in colony counts below the limit of detection. Altogether, these data suggested that *recB* and *recC*, which with *recA* comprise major DSB repair machinery of HR [27,29], exhibit epistasis with *uvrD* in the phenomenon of inhibition-assisted recovery of FQ persisters.

### 2.3. *recA* and *uvrD* are more critical to FQ persister recovery than other NER and HR genes

**Fig 6:**
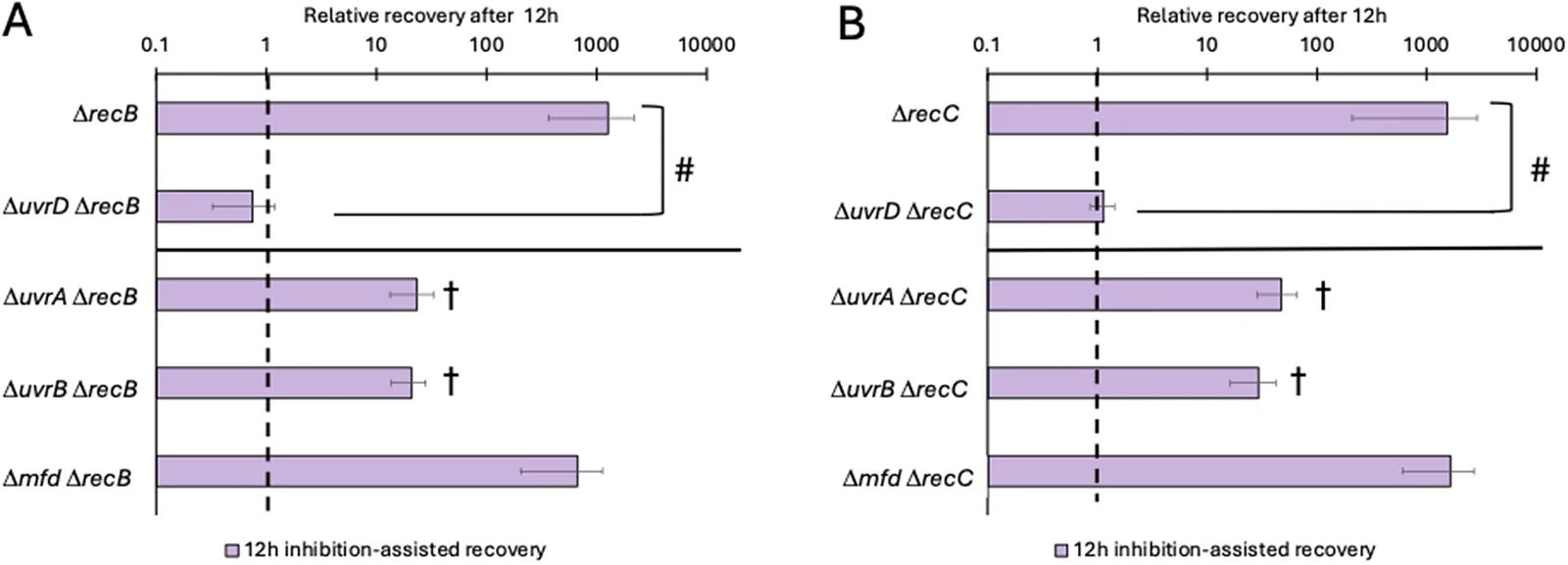
Screen for epistatic interactions of gene combinations that recapitulated the phenotypes of either *recA* or *uvrD* in FQ persister recovery. Stationary-phase cultures of strains harboring indicated mutations were treated with 5 µg/mL OFL and assayed for recovery by washing three times with PBS and plating on LB agar supplemented with 25 µg/mL CAM on top of 0.2 µm filters. After 12 h, filters were transferred to LB agar plates for 24 h. Δ*uvrA* Δ*recB*, Δ*uvrB* Δ*recB* **(A)**, and Δ*uvrA* Δ*recC*, Δ*uvrB* Δ*recC* **(B)** strains exhibited an attenuated response to inhibition-assisted recovery, whereas Δ*mfd* Δ*recB* **(A)** and Δ*mfd* Δ*recC* **(B)** did not exhibit epistasis. Strains here contained kanamycin resistance cassettes from the knockout procedure (S1 Table). Data points reflect the mean values of at least three biological replicates with error bars indicating the standard errors of the means. One-way ANOVA with post-hoc Tukey tests were conducted to assess statistical significance. †: indicates statistical significance (p ≤ 0.05) when compared to single mutants (Δ*recB* or Δ*recC*) and double mutants (Δ*uvrD* Δ*recB* or Δ*uvrD* Δ*recC*). #: reflects statistically significant difference between the indicated references strains.

From the above genetic analyses, we identified that inhibition-assisted recovery of FQ persisters required *mfd*, *uvrA*, *uvrB*, and *uvrD* in strains devoid of *recA*; and *recA*, *recB*, and *recC* were required in strains devoid of *uvrD*. To further delineate interactions in this epistatic network, we generated the remaining double knockout mutants (Δ*uvrA* Δ*recB*, Δ*uvrB* Δ*recB*, Δ*mfd* Δ*recB*, Δ*uvrA* Δ*recC*, Δ*uvrB* Δ*recC*, Δ*mfd* Δ*recC*) and assayed them for FQ persister recovery. For combinations of Δ*mfd* with Δ*recB* or Δ*recC*, FQ persister recovery was still observed (Fig 6), which differed from results with Δ*mfd* Δ*recA* where FQ persister recovery was eliminated (Figs 1 and 3). Alternatively, combinations of Δ*uvrA* with Δ*recB* or Δ*recC* or Δ*uvrB* with Δ*recB* or Δ*recC* exhibited attenuation of inhibition-assisted recovery (Fig 6). These results suggested that epistasis in persister recovery is not simply explainable by epistasis between NER and HR DSB machinery, but rather *recA* and *uvrD* appear to be more critical for FQ persister recovery than *recB*, *recC*, *uvrA*, *uvrB*, and *mfd*.

### 2.4. RecA recombination activity and UvrD helicase activity are needed for FQ persister recovery

**Fig 7:**
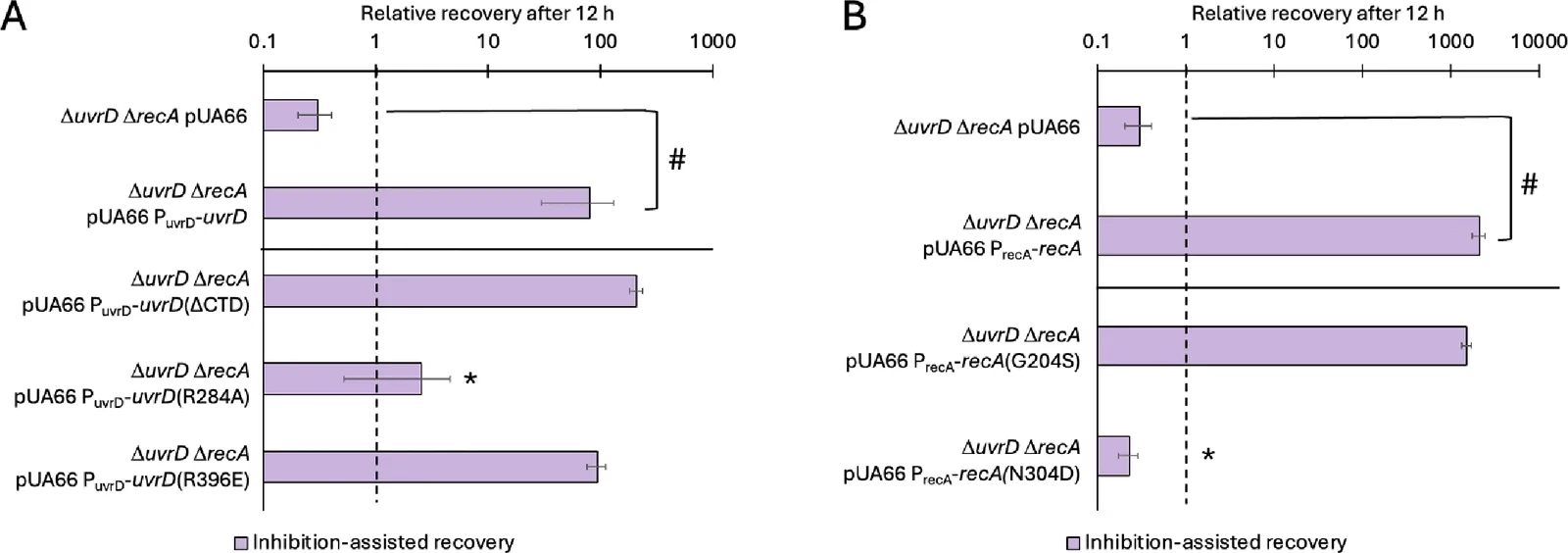
Assessment of separation of function mutants of RecA and UvrD in FQ persister recovery. Stationary-phase cultures of strains harboring plasmids with indicated mutant were treated with 5 µg/mL OFL and assayed for recovery by washing three times with PBS and plating on LB agar supplemented with 25 µg/mL CAM and 50 µg/mL kanamycin on top of 0.2 µm filters. After 12 h, filters were transferred to LB agar plates with kanamycin for 24 h. Mutants of UvrD along with empty vector and native UvrD controls **(A),** and mutants of RecA along with empty vector and native RecA controls **(B).** Data points reflect the mean values of at least three biological replicates with error bars indicating the standard errors of the means. One-way ANOVA with post-hoc Tukey tests were conducted to assess statistical significance. *: reflects statistically significant differences (p ≤ 0.05) between the indicated mutant and native UvrD or RecA, but not empty vector control. #: reflects statistically significant difference between the indicated references strains.

Due to their importance to FQ persister recovery and the fact that both RecA and UvrD have multiple functions, we used a panel of characterized mutants to assess the role of different RecA and UvrD functions in persister recovery. For UvrD, we considered UvrD(R284A), which was shown to eliminate helicase activity while preserving DNA binding activity [48]; UvrD(R396E), which was shown to reduce dsDNA binding activity while preserving helicase activity and ssDNA binding [49]; and UvrD(ΔCTD), which corresponds to deletion of the C-terminal domain (amino acids 645–720), which was shown to reduce the binding of UvrD to RNA polymerase [50]. As depicted in Fig 7A, mutants with reductions in dsDNA binding or RNA polymerase binding were able to restore persister recovery of Δ*uvrD* Δ*recA* to levels that were comparable and statistically indistinguishable from native UvrD. However, UvrD(R284A) failed to restore persister recovery to Δ*uvrD* Δ*recA* and was statistically indistinguishable from the empty vector control. These data suggest that the helicase activity of UvrD was involved in the recovery of FQ persisters, whereas the dsDNA and RNA polymerase binding activities appear to be dispensable functions for the phenomenon. Notably, UvrD(ΔCTD) exhibits a 2-fold reduction in RNA polymerase binding affinity and a significant reduction in UvrD-dependent RNA polymerase backtracking [50,51], which are important interactions for UvrD-mediated TCR [50,52]. The observation that inhibition-assisted recovery with UvrD(ΔCTD) resembles that of native UvrD thereby suggests that UvrD-mediated TCR is not required for the recovery of FQ persisters.

For RecA, we considered RecA(G204S), which is also known as RecA430 and has retained much of its recombination activity while exhibiting little ability to initiate the SOS response [53], and RecA(N304D), which is recombination deficient, but proficient at cleaving LexA and initiating the SOS response [53]. As illustrated in Fig 7B, RecA(N304D) cannot restore persister recovery in Δ*uvrD* Δ*recA*, whereas RecA(G204S) restores persister recovery to levels equivalent to that of native RecA. These results suggest that the recombination functions of RecA provide the basis of the epistatic interaction between RecA and UvrD in the recovery of FQ persisters, whereas the ability of RecA to initiate the SOS response could be a dispensable function, which agrees with the results of *lexA3* in Fig 4.

### 2.5. Loss of DSB and SSG repair machinery do not recapitulate loss of *recA* in inhibition-assisted recovery

**Fig 8:**
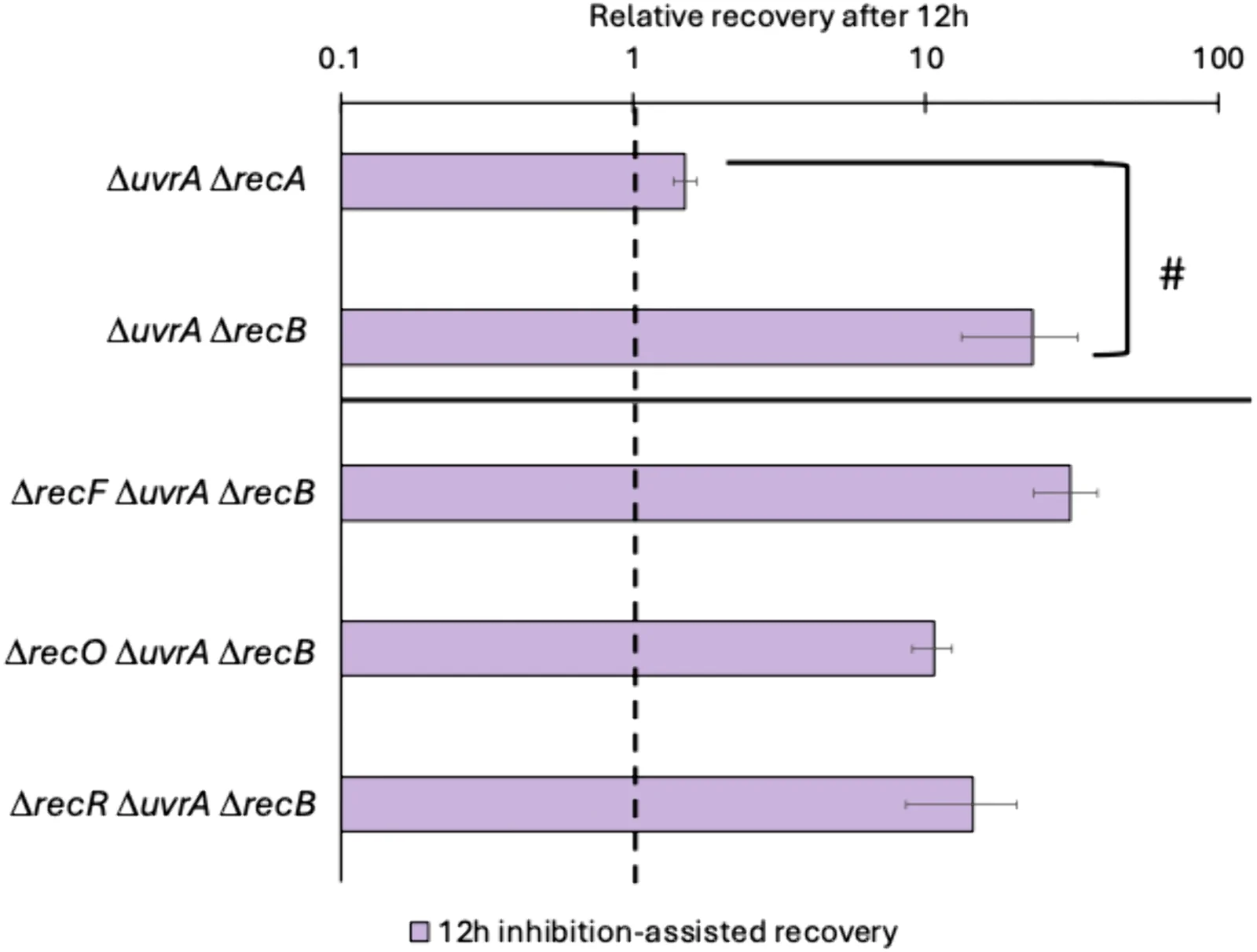
Screen for epistatic interactions of strain combinations deficient in NER, DSB repair, and SSG repair. Stationary-phase cultures of strains harboring indicated mutations were treated with 5 µg/mL OFL and assayed for recovery by washing three times with PBS and plating on LB agar supplemented with 25 µg/mL CAM on top of 0.2 µm filters. After 12 h, filters were transferred to LB agar plates for 24 h. Strains here contained kanamycin resistance cassettes from the knockout procedure (S1 Table). Data points reflect the mean values of at least three biological replicates with error bars indicating the standard errors of the means. One-way ANOVA with post-hoc Tukey tests were conducted to assess statistical significance. None of the triple mutants were statistically different from Δ*uvrA* Δ*recB* and equivalent to Δ*uvrA* Δ*recA*, which would constitute an absence of recovery; and none of the triple mutants were statistically different from Δ*uvrA* Δ*recA* and Δ*uvrA* Δ*recB*, which would constitute an intermediate recovery between the responses of those strains. #: reflects statistically significant difference between the indicated references strains.

To investigate the role of RecA recombination in persister recovery more deeply, we considered that RecA contributes to both DSB repair with *recB* and *recC* and SSG repair with the RecFOR pathway [27,45]. We reasoned that loss of DSB repair and SSG repair would more closely resemble loss of recombination in Δ*recA* because both types of damage repair involve HR. When mutants that were defective in NER (Δ*uvrA*), DSB repair (Δ*recB*), and SSG repair (Δ*recF*, Δ*recO*, or Δ*recR*) were assayed, FQ persister recovery was found to be equivalent to that of Δ*uvrA* Δ*recB* and significantly greater than that of Δ*uvrA* Δ*recA* (Fig 8). These data suggest that loss of DSB repair via Δ*recB* and SSG repair via Δ*recF*, Δ*recO*, or Δ*recR* fails to recapitulate the phenotype of Δ*recA* in FQ persister recovery.

### 2.6. Rifampicin exhibits inhibition-assisted recovery that also depends on *recA* and *uvrD*

**Fig 9:**
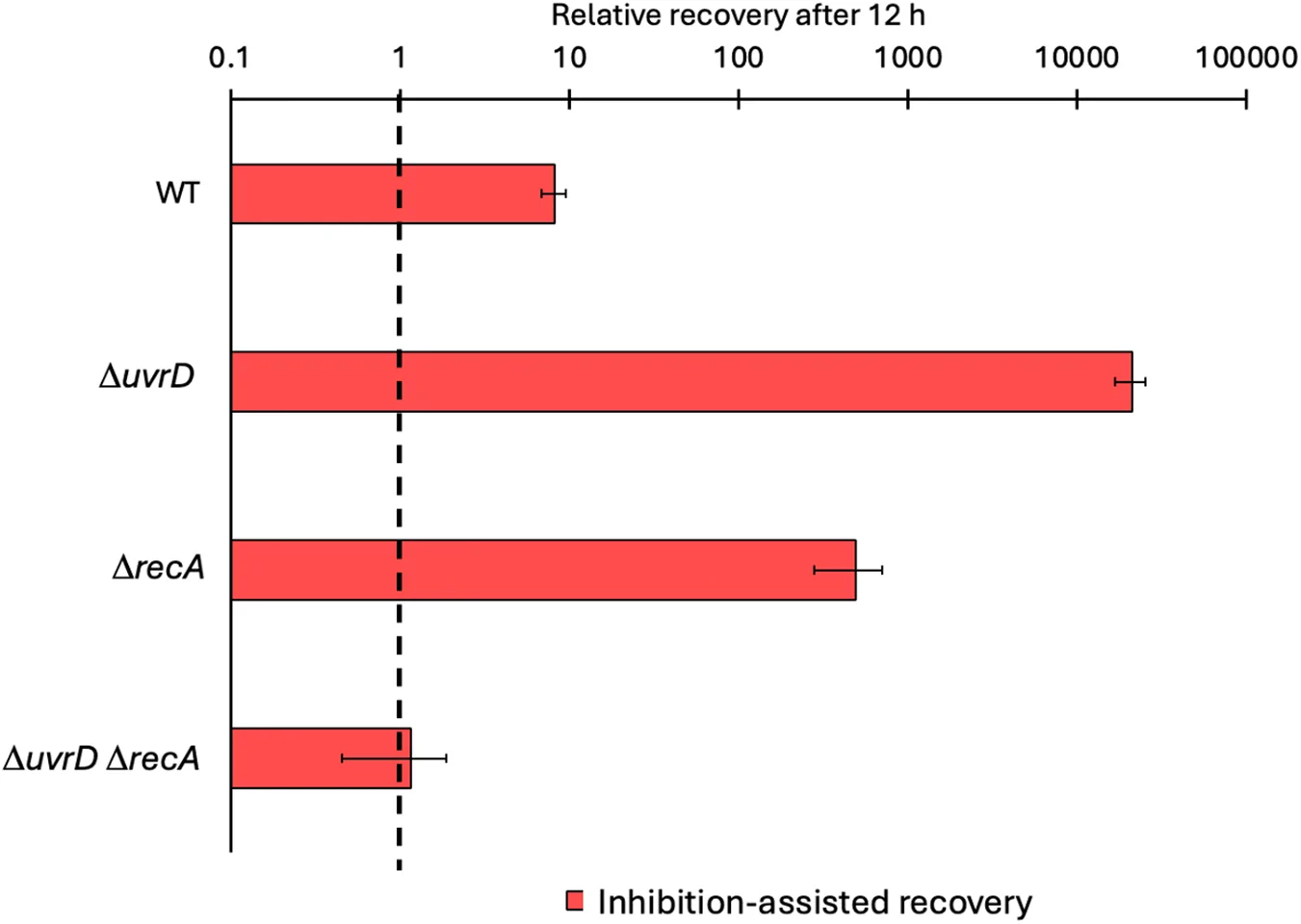
FQ persister recovery with RIF exhibits epistatic interaction with *recA* and *uvrD.* Stationary-phase cultures of strains harboring indicated mutations were treated with 5 µg/mL OFL and assayed for recovery by washing three times with PBS followed by resuspension in LB with 750 µg/mL RIF. Filters were not used due to carryover of RIF, and liquid equivalency was established in previous work (see Materials and Methods). After 12 h, samples were washed and plated on LB agar for 24 h. Strains in these assays were cured of kanamycin resistance cassettes (S1 Table). Data points reflect the mean values of at least three biological replicates with error bars indicating the standard errors of the means.

In previous work, we observed that CAM or rifampicin (RIF) could produce inhibition-assisted recovery of FQ persisters in WT populations [23]. Given the roles of *uvrA*, *uvrB*, *uvrD*, and *mfd* in TCR we sought to determine whether inhibition-assisted recovery had different genetic dependencies when growth inhibition was triggered at the level of transcription (RIF), rather than translation (CAM). Conceivably, if TCR was solely required for persister recovery in the absence of *recA*, the use of RIF would recapitulate the phenotype of Δ*uvrA*, Δ*uvrB*, Δ*uvrD*, or Δ*mfd* when combined with Δ*recA*. As depicted in Fig 9, inhibition-assisted recovery was observed with WT, Δ*uvrD*, and Δ*recA*, but recovery was not observed with Δ*uvrD* Δ*recA*. This genetic dependency mirrors that of recovery with CAM and suggests that TCR is not solely required for FQ persister recovery in Δ*recA*. Rather, results suggest that under conditions with active transcription (CAM used for recovery) Mfd-dependent TCR is required for FQ persister recovery in Δ*recA* (Fig 3), whereas under conditions without transcription (RIF used for recovery) GGR likely provides the analogous function for FQ persister recovery in Δ*recA* (Fig 9).

## 3. Discussion

While persistence to antibiotics had previously been attributed to a lack of antibiotic-induced damage due to cell dormancy, several studies have shown that persisters accumulate DNA damage from FQ treatment, which they have to repair to survive [10,18–26,54]. Our group found that both stationary-phase susceptible cells and OFL persisters experienced DNA damage, which persisters could repair after the conclusion of treatment using DNA repair systems involving RecA [18]. Further, we reported that surviving persisters activated the SOS response and underwent filamentation prior to resuming replication following FQ-induced damage, a response that was highly dependent on RecA [10]. Similarly, Goormaghtigh and Van Melderen showed that this response was conserved in exponential-phase cells, as OFL persisters also experienced DNA damage, and, after treatment, underwent filamentation and induced the SOS response [20]. Later, we discovered that persisters containing two chromosome copies were more likely to survive FQ treatment than one chromosome cells due to their ability to participate in HR DSB repair, as the persister levels in cells with one and two chromosomes were near equivalent in Δ*recA* and Δ*recB* strains [22]. Notably, one chromosome cells still formed persisters, though their growth dynamics differed from both two chromosome persisters and untreated one chromosome controls, suggesting the involvement of HR-independent pathways in the survival of one chromosome cells [22]. Indeed, our group found that RecA was particularly important for the survival of two chromosome FQ persisters, whereas UvrD was important for the survival of one chromosome cells [54]. Further, Wilmaerts and coworkers found that persisters accumulate oxidative damage in response to OFL treatment, and that this damage can be fixed via NER [25]. Combined, these studies show that FQ persisters actively repair antibiotic-induced DNA damage through pathways that include both HR and NER.

Previous studies have suggested that the period after FQ treatment is critical for stationary-phase persister survival [19,23,38]. For example, our group showed that starving *E. coli* cells after FQ treatment resulted in increased persister levels and that this increased survival depended on the presence of RecA [19]. A similar response has also been observed in *Salmonella enterica* cultures where survival increased ~5-fold when cells were exposed to a nutrient-poor environment after FQ treatment compared to immediate exposure to nutrient-rich conditions [38]. Further, this phenomenon is not exclusive to starvation, as delaying growth resumption after FQ treatment either by the accumulation of intracellular toxins, such as MazF and LdrD, or by exposing cells to translational or transcriptional inhibitors, such as RIF or CAM, also resulted in higher persister levels [23]. Specifically, we showed that inhibiting translation following FQ treatment resulted in increased survival in Δ*recA* and Δ*uvrD* strains, but not in the double mutant (Δ*uvrD* Δ*recA*), providing further evidence for the role of HR and NER in FQ persistence [23]. Here, we took inspiration from that previous study and further investigated the epistatic interactions between DNA repair machinery that underlie the ability of persisters to recover from FQs when translation is impaired following treatment.

Using a genetic approach, we found that *uvrD*, *uvrA*, *uvrB*, and *mfd* exhibit epistasis with *recA* during inhibition-assisted recovery (Figs 1–3). These genes, along with *uvrC*, are known to participate in NER, which can be divided into three sub-pathways (GGR, Mfd-mediated TCR, and UvrD-mediated TCR) [32,33,36,37]. Importantly, all NER pathways repair damage on one strand of DNA and are not associated with repair of DSBs, which are discussed below. In GGR, DNA lesions are recognized by changes in helix rigidity [32–34]. In Mfd-mediated TCR, Mfd pushes forward an RNA polymerase stalled at a damage site to expose the lesion, whereas in UvrD-mediated TCR, the lesion is exposed when UvrD promotes backtracking of the stalled RNA polymerase [32,33]. In all three pathways, DNA lesions are recognized by the UvrA_2_B_2_ complex, which scans the genome for helix distortions [26,27]. Specifically, UvrA initiates contact at the damaged site and facilitates loading of UvrB onto the lesion [26,27]. Upon lesion identification, UvrA dissociates, allowing UvrB to recruit UvrC [26,27]. UvrC introduces two incisions, the first on the 3′ side and the second on the 5′ side of the lesion [32,33,35]. UvrD then uses its helicase activity to help remove the damaged nucleotides, and the resulting SSG is filled by DNA polymerase I and sealed by ligase [32,33]. Interestingly, neither UvrC nor Cho, the nucleases associated with NER, were required for inhibition-assisted recovery, although removal of both in Δ*recA* led to an attenuation in persister recovery (Fig 1) [35]. Notably, a prior study by Wilmaerts and colleagues found UvrA, UvrB, UvrD, and Mfd to be important for FQ persister survival, but not UvrC [25]. However, that study did not examine the role of Cho and was not investigating survival changes in populations exposed to a growth-inhibited environment immediately after treatment [25]. We postulate that another nuclease that has not been associated with canonical NER functions substitutes for UvrC and Cho in this process when they are absent. Identifying such a nuclease and how it interacts with canonical NER machinery represents an interesting area for future work. In addition, we observed that transcriptional inhibition after FQ exposure produced persister recovery with the same epistatic interactions between *recA* and *uvrD* that translational inhibition did (Fig 9). Such data suggested that transcription is not required to observe FQ persister recovery in Δ*recA*, and that TCR is not solely responsible for persister recovery in the absence of HR.

To further identify genes in this epistatic network, we implemented a complementary approach to identify *uvrD* epistatic interaction partners. We found that *recA, recB,* and *recC* exhibited epistasis with *uvrD* in inhibition-assisted recovery (Figs 2, 4, and 5), which together with *recD*, repair DSBs through the RecBCD pathway of HR [27,29]. Briefly, the RecBCD helicase activity unwinds duplex DNA and degrades both strands of DNA with its nuclease activity until a Chi site is encountered, which triggers a shift in nuclease activity that leads to the production of a 3’ ssDNA tail [27,29]. The RecBCD complex promotes RecA loading onto that ssDNA, allowing the resulting RecA filament to promote the search for a homologous sequence and strand invasion [27,29]. The involvements of RecB and RecC, but not of RecD, in FQ persistence has been observed previously; however, those studies did not focus on the post-treatment recovery period [25,55]. Several works have reported a less prominent role of RecD in recombination [29,56–60]. Knockout mutants of *recB*, which has both the nuclease domain and 3’ to 5’ helicase activity, or *recC*, which is crucial for Chi site recognition, exhibit reduced viability and recombination deficiency, whereas a ∆*recD* mutation has no effect on viability and cells remain proficient in recombination [29,56–60]. It has been postulated that this may occur due to the ability of RecD to inhibit recombination until encountering a Chi site; thus, Δ*recD* strains would constitutively mimic the post-Chi state, allowing RecBC to perform recombination and repair independently of RecD [29,56–60]. Overall, our results suggest that in the absence of UvrD, persisters can recover from FQ treatment by repairing DSBs through the RecBCD pathway of HR.

The epistatic interactions of *uvrA*, *uvrB*, and *mfd* with *recA,* suggest that persister levels can increase through the repair of ssDNA lesions with the use of NER. Such lesions could result from gyrase monomers or topoisomerase IV subunits that remain covalently bound to the phosphate backbone on one side of DNA, or from the accumulation of oxidative lesions caused by FQ treatment [13–16,25,61]. In contrast, the epistatic interactions of *recB* and *recC* with *uvrD* suggest that persisters survive by repairing DSBs caused by FQ treatment through the RecBCD pathway of HR. Previous work has reported epistatic interactions between HR and NER in the repair of DNA-protein crosslinks (DPCs) [62–64]. NER can repair DPCs smaller than 12–14 kDa [62,63], which could mimic lesions formed by the degradation of *gyrA* or *parC* monomers that had remained bound to DNA following FQ treatment. This restriction reflects the limited ability of UvrB to load onto larger DPCs sites, which is needed to recruit UvrC to excise the damage [62,63]. Conversely, HR can process DPCs across a wider size range, including both small and large lesions [62,63]. Given that HR represents a more versatile system for repairing DNA damage than NER, it could explain why mutants deficient in NER but with intact HR recover faster post-FQ treatment than those deficient in HR but with intact in NER (Figs 2–3 and 5). Importantly, while restoration of culturability was measured here for a variety of DNA repair mutants, measurement of DNA damage in persisters was not feasible. Specifically, persisters are minority populations that are difficult to differentiate from viable but non-culturable cells (VBNCs) prior to outgrowth [65], which makes measurement of DNA damage in them difficult. Conceivably, fluorescent reporters of DNA damage can be used [18]; however, those reporters often rely on the SOS response, which is inactive in most of the strains examined here due to the use of Δ*recA*. Alternatively, population level metrics of DNA damage can be used to assess whether correlations exist between population-wide measurements and persister recovery, such as gyrase cleavage site sequencing (GCS-seq) [55] and bacterial rapid approach to DNA adduct recovery (RADAR) [66]. In the future, application of such techniques to the strains examined here could identify whether differential levels of DNA damage underlie any of the trends observed; however, given that the OFL concentration used here was ~ 80-fold the MIC of WT [67], it is likely that any difference in DNA damage detected would reflect differences in ability to repair the damage or differences in DNA gyrase or topoisomerase activity between strains.

To delineate additional dependencies in the epistatic interaction network underlying FQ persister recovery, we generated the remaining double knockout mutant combinations between the NER and HR machinery that contribute to the recovery of FQ persisters (Δ*uvrA* Δ*recB*, Δ*uvrB* Δ*recB*, Δ*mfd* Δ*recB*, Δ*uvrA* Δ*recC*, Δ*uvrB* Δ*recC*, Δ*mfd* Δ*recC*), all of which retained functional RecA and UvrD. Unexpectedly, none of those combinations completely recapitulated the absence of inhibition-assisted recovery observed in strains devoid of *recA* or *uvrD* (Fig 6). Given that HR can proceed through the RecFOR pathway to repair SSGs [27,45], we constructed triple knockout mutants defective in SSG repair, DSB repair, and NER (Δ*recF* Δ*uvrA* Δ*recB,* Δ*recO* Δ*uvrA* Δ*recB,* Δ*recR* Δ*uvrA* Δ*recB*) while retaining functional RecA and UvrD. Loss of both HR pathways through combined deletions of Δ*recB* and Δ*recF,* Δ*recO,* or Δ*recR* still failed to recapitulate the phenotype of Δ*recA* (Fig 8). Likewise, loss of Δ*uvrA*, Δ*uvrB*, and Δ*mfd* did not recapitulate loss of *uvrD*. Together, these findings suggest that inhibition-assisted recovery depends on RecA- and UvrD-mediated activities that go beyond simply a loss of HR or NER in their respective deletion mutants. To elucidate the functions of RecA and UvrD that were required for inhibition-assisted recovery we used a panel of mutants where selective functions of RecA and UvrD had been knocked down. Those experiments revealed that the recombination activity of RecA and helicase activity of UvrD were important to FQ persister recovery, whereas reductions in SOS induction by RecA and dsDNA and RNA polymerase binding by UvrD did not impact the phenomenon (Fig 7).

Beyond the prototypical role of UvrD in NER, studies have suggested a role for it outside of NER [63,68–70]. As Δ*uvrD* strains exhibit elevated levels of HR, it has been proposed that UvrD helps remove RecA filaments from stalled replication forks, which prevents unnecessary recombination that could otherwise be lethal to the cell [68–70]. Additionally, *uvrD* mutants showed modest sensitivity to azacytidine, which induces DPCs that exceed the size limit for NER and are therefore expected to be processed exclusively by HR [62,63]. Beyond the prototypical role of RecA in HR, Buljubašić and colleagues described a third HR pathway active in Δ*recBCD sbcB15 sbcC*(*D*) Δ*recF*(*OR*) mutant derivatives that enables efficient DNA repair independent of RecBCD and RecFOR, but dependent on RecA and the SbcB15 allele [71]. In that pathway, the RecQ helicase unwinds DNA and the RecJ exonuclease produces a 3’ ssDNA overhang where RecA can bind [71]. Exploring the additional interactions of *uvrD* and *recA* that give them higher degrees of epistasis in this phenomenon are interesting areas of future study.

**Fig 10:**
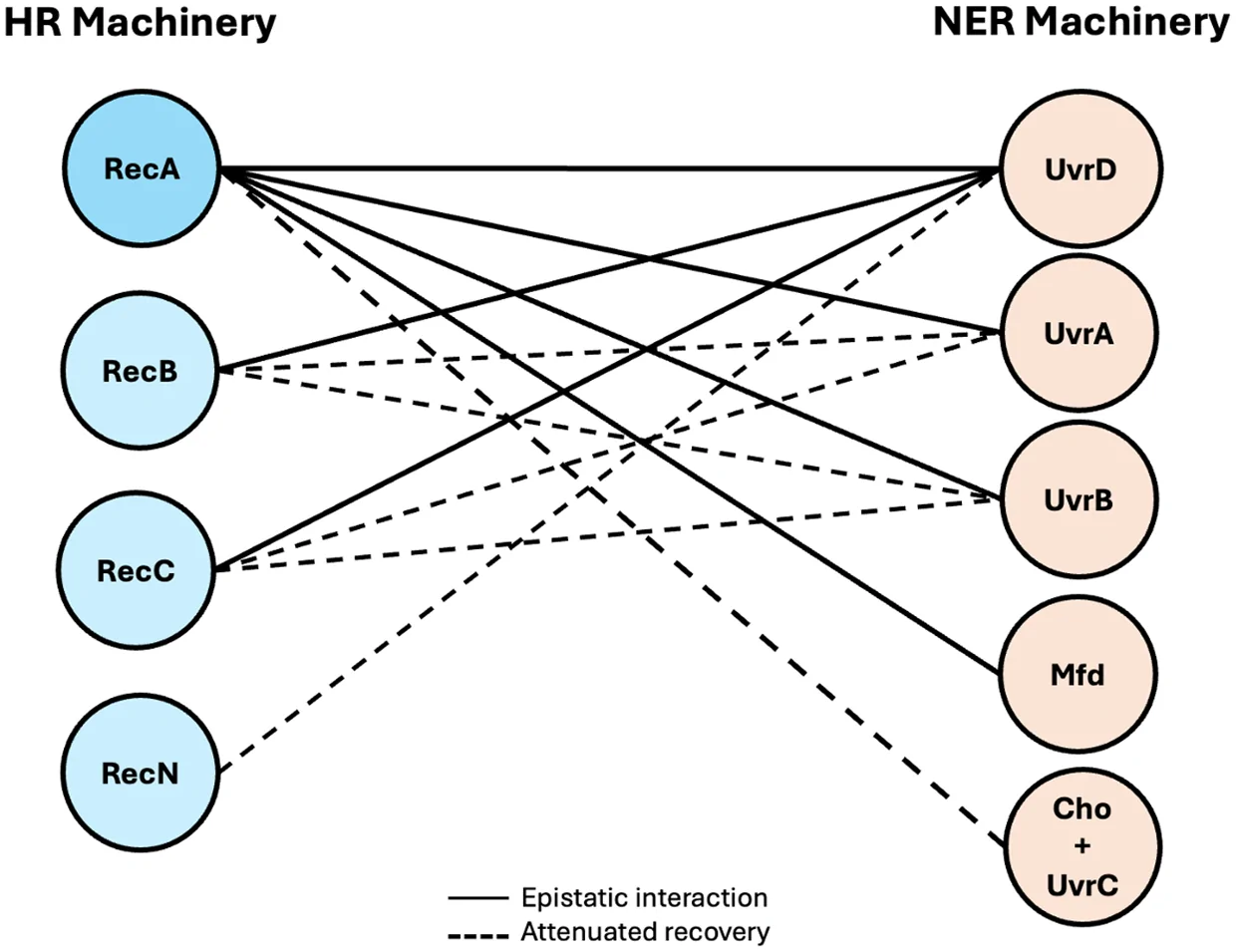
Diagram of the epistatic interaction network driving FQ persister recovery. Solid lines indicate epistatic interactions of *uvrA*, *uvrB*, and *mfd* with *recA*, and epistatic interactions of *recB* and *recC* with *uvrD.* Dashed lines indicate attenuated recovery levels for the Δ*uvrD* Δ*recN* and Δ*cho* Δ*uvrC* Δ*recA* mutants, as well as when *uvrA* or *uvrB* were deleted alongside either *recB* or *recC*.

Collectively, the results presented here suggest that HR machinery facilitated FQ persister recovery that was observable in the immediate hours after FQ treatment, whereas the contributions of NER machinery became apparent at longer timescales in the absence of RecA. Further, combinations of NER and HR DSB knockout mutants failed to fully recapitulate the phenotypes of *uvrD* or *recA,* which suggested the existence of an epistatic network where RecA and UvrD had stronger connections to other nodes than other members (Fig 10). In addition, we determined that the recombination activity of RecA and helicase activity of UvrD were essential to their epistatic interaction in this network. Overall, this study elaborates on an epistatic interaction network involving HR and NER machinery in the recovery persisters from FQ treatment and further elaborates on the critical roles that RecA and UvrD in this phenomenon.

## 4. Materials and Methods

### 4.1. Bacterial strains and plasmids

All strains used in this study were derived from *E. coli* MG1655, and details about their construction are provided in S1 Table. Genetic knockouts were transduced from the respective Keio collection mutant via P1 phage transduction [72,73]. When indicated, kanamycin resistance markers were removed using FLP recombinase expressed from pCP20 or pGL04 [74,75]. Knockouts were confirmed via PCR with primers that anneal to the gene of interest (internal primer pair), the upstream and downstream sequences of the gene of interest (external primer pair), and upstream to the gene and internal to the kanamycin resistance cassette. All primer sequences are provided in S2 Table. The uncleavable *lexA* mutant, *lexA3*, was constructed in a previous study [24], and the mutation was confirmed by whole genome sequencing (Plasmidsaurus Inc.).

For complementation of *recA, uvrA, uvrB, mfd, uvrD*, *recB,* and *recC*, pUA66 plasmids containing either P_recA_-*recA*, P_uvrA_-*uvrA,* P_uvrB_-*uvrB*, P_mfd_-*mfd,* P_uvrD_-*uvrD*, P_recB_-*recB*, or P_recC_-*recC* were used. These plasmids were constructed using Gibson Assembly [76], verified by whole plasmid sequencing (Plasmidsaurus Inc.), and transformed into strains of interest by electroporation (see below). A pUA66 plasmid containing a promoterless *gfp*_*mut2*_ was transformed into our strains of interest and used as an empty vector control. For chromosomal complementation at the Tn7 attachment site (*attTn7*), transpositions were conducted using pGRG25 as described previously [47]. A fragment containing P_uvrD_-*uvrD* was introduced into the MCS of pGRG25 via Gibson assembly [76] and confirmed by whole plasmid sequencing (Plasmidsaurus Inc.). The non-coding MCS between the left and right transposition arms of pGRG25 was used as an empty control. Plasmids were transformed into Δ*uvrD* Δ*recB* and Δ*uvrD* Δ*recC* by electroporation. Transpositions of interest were confirmed via PCR with primers that flank the *attTn7* site listed in S2 Table, and loss of pGRG25 was confirmed by the absence of growth in ampicillin at 30–32 ℃.

Separation-of-function mutations were introduced into plasmids harboring the WT sequence, pUA66 P_recA_-*recA* or pUA66 P_uvrD_-*uvrD*, using the Q5 Site-Directed Mutagenesis Kit (New England Biolabs). Briefly, plasmid backbones were amplified with mutagenic primers using the provided Q5 Hot Start High-Fidelity 2X Master Mix. Following amplification, products were treated with the provided Kinase-Ligase-DpnI (KLD) enzyme mix at room temperature for 5 min. All mutations were confirmed via whole plasmid sequencing (Plasmidsaurus Inc.), and plasmids were transformed into Δ*uvrD* Δ*recA* by electroporation. All plasmids used in this study are listed in S3 Table, and the primers used for plasmid construction can be found in S4 Table.

To generate electrocompetent cells, strains of interest were first inoculated from -80 °C, 25% glycerol stocks into 3 mL of LB media and incubated at 37 °C with shaking at 250 r.p.m for at least 16 h. Overnight cultures were then diluted 100-fold in 3 mL of SOB media. When cultures reached mid-exponential phase (OD_600_ = ~0.4-0.6), 1 mL samples were added to microcentrifuge tubes and chilled on ice for 10 min. Chilled cultures were washed three times. In each wash, cells were collected by centrifugation at 5,000 r.p.m for 5 min at 4 ºC, followed by removal of supernatants. In the first wash, cell pellets were resuspended in 1 mL of ice-chilled 1% glycerol. In the second and third washes, cell pellets were resuspended in 1 mL of ice-chilled 10% glycerol. After the third wash, cell pellets were resuspended in 0.1 mL of ice-chilled 10% glycerol to produce electrocompetent cells. For electroporation, electrocompetent cells were mixed with ~100 ng of purified plasmid and chilled on ice for 30 min. The mixture was then added to an electroporation cuvette and electroporated. Afterward, 900 μL of SOC media was added to cuvettes and mixtures were transferred to microcentrifuge tubes. Cultures were then incubated at 37 ºC (for pUA66 or pGL04 transformations) or 30–32 ºC (for pGRG25 or pCP20 transformations) for 1 h, after which they were centrifuged at 15,000 r.p.m for 3 min. After incubation, 900 μL of supernatants were removed and the remaining culture volume was plated on LB agar containing the appropriate selection marker.

### 4.2. Chemicals and media

All chemical components were obtained from Fisher Scientific or Sigma Aldrich, unless otherwise specified. All media were prepared in distilled water purified using a Millipore Milli-Q lab water system (Burlington, MA) to a resistivity of 18.2 MΩ.cm. LB media was made by dissolving 10 g/L tryptone, 5 g/L yeast extract, and 10 g/L NaCl in MilliQ water, which was then autoclaved. LB agar plates were made with 25 g/L pre-mixed LB Miller broth and 15 g/L agar dissolved in MilliQ water, which was autoclaved. For P1 transduction, special LB medium was made by adding 1.75 mL of 1 M CaCl_2_ and 3 mL of 1 M MgSO_4_ 250 mL of LB media, and for mutant selection sodium-citrate plates were made by supplementing LB agar plates with 25 mM of sterile-filtered sodium citrate and kanamycin. For bacterial transformations, SOB media was prepared by autoclaving 20 g/L tryptone, 5 g/L yeast extract, 5 mM NaCl (5 M filter sterilized stock solution), and 2.5 mM KCl (1 M filter sterilized stock solution) in MilliQ water. After, a 1 M MgSO_4_ stock solution was prepared in MilliQ water, filter-sterilized, and added to a final concentration of 10 mM. SOC media was prepared by supplementing SOB media with 20 mM of filter-sterilized 1 M glucose. To generate electrocompetent cells and cells stocks, 1%, 10%, and 50% glycerol solutions were prepared by diluting the appropriate amount of glycerol in autoclaved water and sterilizing with 0.22 μM bottle top filters (Merck Millipore Ltd, Burlington, MA). For curing of pGL04, no salt LB plates were prepared by dissolving 1 g tryptone, 0.5 g yeast extract, and 1.5 g agar in 80 mL MilliQ water and autoclaving. Once that mixture was cool to touch, 20 mL of 1.5 M filter-sterilized sucrose was added. To induce the expression of *tns*ABCD from pGRG25, 1 M stocks of L-arabinose were prepared in autoclaved water and filter sterilized. Gutnick media used for persister and recovery assays was made by mixing 10 mM glucose (filter sterilized), 10 mM NH_4_Cl (filter sterilized), and 1X Gutnick salts in autoclaved MilliQ water, and sterilizing with 0.22 μM bottle top filters. Autoclaved 10-fold concentrated (10X) Gutnick salts solution contained 47 g/L KH_2_PO_4_, 135 g/L K_2_HPO_4_, 10 g/L K_2_SO_4_, and 1 g/L MgSO_4_·7H_2_O in MilliQ water. For all wash steps, sterile-filtered 1X phosphate-buffered saline (PBS) solution prepared from a 10X stock was used. The 10X stock was prepared by mixing 98.9 g of powdered PBS (81% NaCl, 14% Na_2_HPO_4_, 3% KH_2_PO_4_, and 2% KCl by weight) in 1 L of Milli-Q water followed by autoclaving.

For mutant selection and plasmid retention, a final concentration of 100 μg/mL ampicillin or 50 μg/mL kanamycin were used in media and plates. For FQ persister assays, 5 μg/mL OFL was used. For translational inhibition, a final concentration of 25 μg/mL CAM was added to autoclaved LB agar. Stock solutions for all antibiotics and chemicals, except CAM, RIF, and OFL, were prepared in autoclaved MilliQ water and filter sterilized before use. CAM was dissolved in ethanol, RIF was dissolved in DMSO, and 1M NaOH was added to OFL stock solution in autoclaved MilliQ water until it was fully dissolved and then filter sterilized.

### 4.3. Persistence assays

Cultures were first inoculated from -80 °C, 25% glycerol stocks into 3 mL of LB media and incubated at 37 °C with shaking at 250 r.p.m. After 4 h, those cultures were diluted 100-fold in 3 mL of Gutnick media with 10 mM glucose as the sole carbon source and incubated at 37 °C with shaking at 250 r.p.m. for 20 h. After 20 h of incubation, 500 µL samples were removed for t = 0 h measurements prior to antibiotic treatment. Then OFL was added to a final concentration of 5 µg/mL, cultures were incubated at 37 °C with shaking at 250 r.p.m. and additional 500 µL samples were taken after 1, 3, and 5 h. For all time points, samples were washed three times by centrifugation at 15,000 r.p.m for 3 min, removal of 450 µL of supernatant, and resuspension of cell pellets in 450 µL of sterile PBS. After the three washes, samples were spun once more and concentrated 5-fold by removing 400 µL of supernatant and resuspending the cell pellet in the remaining 100 µL of PBS. Ten µL of the concentrated samples were used for 10-fold serial dilutions in 90 µL of PBS. For each sample, 10 µL per dilution were spotted onto LB agar plates that were incubated at 37 °C for 24 h, after which CFUs were enumerated for dilutions that were countable and contained at least 5 colonies.

### 4.4. Recovery assays

After 5 h of OFL treatment, 500 µL samples were taken and washed three times with sterile PBS. Each wash step consisted of a centrifugation step at 15,000 r.p.m for 3 min, removal of 450 µL of supernatant, and resuspending pellets in 450 µL of PBS. After the three washes, samples were spun once more and concentrated 5-fold by removing 400 µL of supernatant and resuspending cells in the remaining 100 µL of PBS. Ten-fold serial dilutions in PBS were made for each sample. For t = 0 h, a 10 µL spot of each dilution was plated directly onto an LB agar plate. For all other timepoints, 10 µL of each dilution were spotted onto Supor 200 polyether sulfone membranes with 0.2 µm pores (Pall Corporation) that were placed atop of LB agar plates supplemented with 25 µg/mL CAM and incubated at 37 °C. As shown previously, membranes were used to immobilize cells to ensure that CFU counts were not influenced by any cell divisions that occurred during the recovery period [19,23]. After 2, 4, 6, 8, 10, or 12 h, membranes were transferred to LB agar plates and incubated at 37 °C for 24 h, after which CFUs were counted for dilutions that were countable and contained at least 5 colonies. For initial mutant screening, membranes were transferred to LB agar plates only at t = 12 h. Agar thickness was doubled for LB plates receiving membranes from CAM-supplemented plates to support bacterial growth, as it allowed any residual antibiotic from the membrane to disperse.

For RIF recovery assays, use of filters was not possible due to the affinity of the filters for RIF [23]. Therefore, liquid based assays were used, which were shown previously to be indistinguishable from filter-based assays using CAM [23]. In brief, after 5 h of OFL treatment, 500 µL samples were taken and washed three times with sterile PBS. Each wash step consisted of a centrifugation step at 15,000 r.p.m for 3 min, removal of 450 µL of supernatant, and resuspension of pellets in 450 µL of PBS. After three washes, samples were spun once more and concentrated 5-fold by removal of 400 µL of supernatant and resuspension of cells in the remaining 100 µL of PBS. Cells were then resuspended in liquid LB supplemented with 750 µg/mL RIF, a concentration previously observed to halt transcription and TCR in *E. coli* [37,77]. Specifically, 30 µL aliquots of the previously mentioned 100 µL cell suspensions were inoculated into 3 mL of liquid LB supplemented with RIF. These cultures were incubated at 37 °C with shaking at 250 r.p.m for 12 h. A 500 µL sample was taken before and after the 12 h incubation. For each time point, samples were washed three times, concentrated 5-fold, and 10-fold serially diluted in PBS. Ten µL per dilution were plated onto LB agar plates and incubated at 37 °C for 24 h. Further, an additional 75 µL of undiluted samples were also plated in the case of low culturability samples. CFUs were enumerated for dilutions that were countable and contained at least 5 colonies.

To confirm that 750 µg/mL of RIF was sufficient to halt bacterial growth under our experimental conditions, control experiments were performed using untreated (no FQ) cultures (S4 Fig). Cells were incubated for a total of 25 h, to account for the standard 20 h overnight growth period and a 5 h mock-treatment period. Following incubation, cultures were processed as described above, with a single modification to account for the absence of FQ-induced cell death. Instead of inoculating a 30 µL aliquot of the 100 µL concentrate, the untreated cultures were diluted to approximately the same survival levels observed in their respective OFL-treated counterparts. Specifically, the WT strain was diluted an additional 20-fold, whereas the mutant strains Δ*uvrD*, Δ*recA*, and Δ*uvrD* Δ*recA* were diluted an additional 5,000-fold, 50,000-fold, and 125,000-fold, respectively.

### 4.5. Statistical analysis

Data points indicate the mean values of at least three biological replicates and error bars represent the standard errors of the means. Where indicated, one way ANOVA with post-hoc Tukey tests were performed to assess statistical significance among the different timepoints of the same condition or between the indicated mutants. Asterisks (*), obelisks (†), and hash marks (#) denote p-values ≤ 0.05.

## Supporting information

S1 Fig Complementation of RecA or UvrD restores inhibition-assisted recovery. Δ*uvrD* Δ*recA* containing pUA66 plasmids with P_uvrD_-*uvrD* or P_recA_-*recA*, or without an expression cassette (empty control) were treated with 5 µg/mL OFL for 5 h. All samples exhibited biphasic killing during OFL treatment, indicating the presence of persisters. For recovery assays, cells were washed with PBS after OFL treatment and plated on filters on top of LB agar with 25 µg/mL of CAM for the designated time before being transferred to LB agar for 24 h. Complementation of *uvrD* or *recA* in the double deletion mutant restored inhibition-assisted recovery, whereas the empty vector control showed no difference in survival. Data points indicate the means of at least three biological replicates with error bars indicating standard errors of the means. One-way ANOVA with post-hoc Tukey test were conducted to assess significance. *: indicates statistically significant (p < 0.05) differences between time points and t = 0 h of the same samples.
(TIF)

S2 Fig Complementation of Δ*uvrA*, Δ*uvrB,* Δ*mfd,* and Δ*recA* restores inhibition-assisted recovery. Δ*uvrA* Δ*recA* containing pUA66 plasmids with P_uvrA_-*uvrA*, P_recA_-*recA*, or without an expression cassette (empty control) (A), Δ*uvrB* Δ*recA* containing pUA66 plasmids with P_uvrB_-*uvrB*, P_recA_-*recA*, or without an expression cassette (empty control) (B), and Δ*mfd* Δ*recA* containing pUA66 plasmids with P_mfd_-*mfd*, P_recA_-*recA*, or without an expression cassette (empty control) (C) were treated with 5 µg/mL OFL for 5 h. All samples exhibited biphasic killing during OFL treatment, indicating the presence of persisters. For recovery assays, cells were washed with PBS after OFL treatment and plated on filters on top of LB agar with 25 µg/mL of CAM for the designated time before being transferred to LB agar for 24 h. Complementation of *uvrA*, *uvrB*, *mfd*, or *recA* in the double deletion mutants restored inhibition-assisted recovery. Empty vector controls showed only small changes in survival. Note that the survival change for the t = 12 h CAM exposure for Δ*uvrA* Δ*recA* with the empty vector was significant but only ~3-fold higher than t = 0 h, whereas the *recA*- and *uvrA*-complemented strains were significant and ~20- and ~40-fold higher, respectively. Also, the timepoints for Δ*uvrB* Δ*recA* with the empty vector that were significantly different than t = 0 h were all lower survival than t = 0 h. Data points indicate the means of at least three biological replicates with error bars indicating standard errors of the means. One-way ANOVA with post-hoc Tukey test were conducted to assess significance. *: indicates statistically significant (p < 0.05) differences between time points and t = 0 h of the same samples. P-values for Δ*uvrB* Δ*recA* containing pUA66 plasmids with P_uvrB_-*uvrB* at t = 8 h and t = 10 h were ~0.064 and ~0.054, respectively.
(TIF)

S3 Fig Complementation of Δ*uvrD* Δ*recB* and Δ*uvrD* Δ*recC* restores inhibition-assisted recovery. Δ*uvrD* Δ*recB* with pUA66 plasmid containing P_recB_*-recB,* P_uvrD_*-uvrD* or without an expression cassette (empty control) and Δ*uvrD* Δ*recB* with a chromosomal insertion at the Tn7 site containing an empty MCS control or P_uvrD_-*uvrD* (A), and Δ*uvrD* Δ*recC* with pUA66 plasmid containing P_recC_*-recC,* P_uvrD_*-uvrD* or without an expression cassette (empty control) and Δ*uvrD* Δ*recC* with a chromosomal insertion at the Tn7 site containing an empty MCS control or P_uvrD_-*uvrD* (B) were treated with 5 µg/mL OFL for 5 h. All samples, except Δ*uvrD* Δ*recC* containing an empty MCS control at the Tn7 site, exhibited biphasic killing during OFL treatment, indicating the presence of persisters. For recovery assays, cells were washed with PBS after OFL treatment and plated on filters on top of LB agar containing 25 µg/mL of CAM for the designated time before being transferred to LB agar for a 24 h incubation at 37 ℃. Plasmid-mediated complementation of *recB* or *recC* in the double deletion mutants and chromosomal complementation of *uvrD* at the *attTn7* site for both double knockout mutants restored inhibition-assisted recovery. Plasmid-mediated complementation of *uvrD* in Δ*uvrD* Δ*recC* showed no significant increases in survival. Since plasmid-mediated empty vector control for Δ*uvrD* Δ*recB* resulted in colony counts that were below the limit of detection, we inserted a MCS at the chromosomal *attTn7* site to use as our empty control, which showed no increase in survival. Both plasmid and chromosomal empty vector controls for Δ*uvrD* Δ*recC* resulted in colony counts that were below the limit of detection either after 1 h of treatment or during the recovery period. Data points indicate the means of at least two biological replicates with error bars indicating the standard errors of the means. One-way ANOVA with post-hoc Tukey test were conducted to assess significance. *: indicates statistically significant (p < 0.05) differences between time points and t = 0 h of the same samples.
(TIF)

S4 Fig RIF treatment inhibits bacterial growth. Stationary-phase cultures of WT or the indicated mutant strains were washed three times with PBS followed by resuspension in LB or in LB supplemented with 750 µg/mL RIF for 12 h. Filters were not used due to carryover of RIF, and liquid equivalency was established in previous work (see Materials and Methods). Before and after the 12 h incubation, samples were washed and plated on LB agar for 24 h. Strains in these assays were cured of kanamycin resistance cassettes (S1 Table). Data points reflect the mean values of at least three biological replicates with error bars indicating the standard errors of the means. *: reflects statistically significant differences (p < 0.05) between the 0 and 750 µg/mL RIF concentrations for the indicated strains.
(TIF)

S1 Table List of strains used in the manuscript. (DOCX)

S2 Table List of primers (5’ → 3’) used to verify genetic modifications. (DOCX)

S3 Table List of plasmids used in this study. (DOCX)

S4 Table List of primers used for plasmid construction. (DOCX)
