# Supplementary material for: Genetic analysis of epistasis between nucleotide excision repair and homologous recombination in the recovery of persisters after fluoroquinolone treatment
Source: PLoS Genet. 2026 Sep 21;22(9):e1012305. doi: 10.1371/journal.pgen.1012305 (PMC13592689; doi:10.1371/journal.pgen.1012305)
Supplement: Supplementary file 1 [file pgen.1012305.s005.docx]

**Supplementary Table 1:** List of strains used in the manuscript.

| **Strain** | **Relevant genotype** | **Source** | **Notes** |
| --- | --- | --- | --- |
| WT MG1655 | F-, λ-, *ilvG*-, *rfb-50, rph-1* | (1) | Original strain from which all other strains are derived. Used for time-course assays. |
| Δ*recA*::*kanR* | MG1655 Δ*recA*::*kanR* | (2) | Generated through P1 transduction using Δ*recA*::*kanR* from the Keio collection (3) as donor into MG1655 recipient. Used for recovery screens. |
| Δ*recA* | MG1655 Δ*recA*::FRT | (2) | Generated by curing Δ*recA*::*kanR* of *kanR* by pCP20 (4), which results in an FRT site in place of the antibiotic resistance cassette. Used for time-course assays. |
| Δ*uvrD*::*kanR* | MG1655 Δ*uvrD*::*kanR* | (2) | Generated through P1 transduction using Δ*uvrD*::*kanR* from the Keio collection (3) as donor into MG1655 recipient. Used for recovery screens. |
| Δ*uvrD* | MG1655 Δ*uvrD*::FRT | (2) | Generated by curing Δ*uvrD*::*kanR* of *kanR* by pCP20 (4), which results in an FRT site in place of the antibiotic resistance cassette. Used for time-course assays. |
| Δ*uvrD* Δ*recA*::*kanR* | MG1655 Δ*uvrD*::FRT Δ*recA*::*kanR* | (2) | Generated through P1 transduction using Δ*recA*::*kanR* as donor into Δ*uvrD*::FRT recipient. Used for recovery screens. |
| Δ*uvrD* Δ*recA* | MG1655 Δ*uvrD*::FRT Δ*recA*::FRT | (2) | Generated by curing Δ*uvrD*::FRT Δ*recA*::*kanR* of *kanR* by pCP20 (4), which results in an FRT site in place of the antibiotic resistance cassette. Used for time-course assays. |
| Δ*uvrA* | MG1655 Δ*uvrA*::FRT | This work | Generated through P1 transduction using Δ*uvrA*::*kanR* from the Keio collection (3) as donor into MG1655, followed by curing of *kanR* by pCP20 (4), which results in an FRT site in place of the antibiotic resistance cassette. Used for time-course assays. |
| Δ*uvrA* Δ*recA*::*kanR* | MG1655 Δ*uvrA*::FRT Δ*recA*::*kanR* | This work | Generated through P1 transduction using Δ*recA*::*kanR* as donor into Δ*uvrA*::FRT recipient. Used for recovery screens. |
| Δ*uvrA* Δ*recA* | MG1655 Δ*uvrA*::FRT Δ*recA*::FRT | This work | Generated by curing Δ*uvrA*::FRT Δ*recA*::*kanR* of *kanR* by pCP20 (4), which results in an FRT site in place of the antibiotic resistance cassette. Used for time-course assays. |
| Δ*uvrB* | MG1655 Δ*uvrB*::FRT | This work | Generated through P1 transduction using Δ*uvrB*::*kanR* from the Keio collection (3) as donor into MG1655, followed by curing of *kanR* by pCP20 (4), which results in an FRT site in place of the antibiotic resistance cassette. Used for time-course assays. |
| Δ*uvrB* Δ*recA*::*kanR* | MG1655 Δ*uvrB*::FRT Δ*recA*::*kanR* | This work | Generated through P1 transduction using Δ*recA*::*kanR* as donor into Δ*uvrB*::FRT recipient. Used for recovery screens. |
| Δ*uvrB* Δ*recA* | MG1655 Δ*uvrB*::FRT Δ*recA*::FRT | This work | Generated by curing Δ*uvrB*::FRT Δ*recA*::*kanR* of *kanR* by pCP20 (4), which results in an FRT site in place of the antibiotic resistance cassette. Used for time-course assays. |
| Δ*uvrC* Δ*recA*::*kanR* | MG1655 Δ*uvrC*::FRT Δ*recA*::*kanR* | This work | Generated through P1 transduction using Δ*uvrC::kanR* from the Keio collection (3) as donor into MG1655 recipient, followed by curing of *kanR* by pCP20 (4), which results in an FRT site in place of the antibiotic resistance cassette. A second round of P1 transduction was conducted using Δ*recA*::*kanR* as donor into Δ*uvrC*::FRT recipient. Used for recovery screens. |
| Δ*mfd* | MG1655 Δ*mfd*::FRT | This work | Generated through P1 transduction using Δ*mfd*::*kanR* from the Keio collection (3) as donor into MG1655, followed by curing of *kanR* by pCP20 (4), which results in an FRT site in place of the antibiotic resistance cassette. Used for time-course assays. |
| Δ*mfd* Δ*recA*::*kanR* | MG1655 Δ*mfd*::FRT Δ*recA*::*kanR* | This work | Generated through P1 transduction using Δ*recA*::*kanR* as donor into Δ*mfd*::FRT recipient. Used for recovery screens. |
| Δ*mfd* Δ*recA* | MG1655 Δ*mfd*::FRT Δ*recA*::FRT | This work | Generated by curing Δ*mfd*::FRT Δ*recA*::*kanR* of *kanR* by pCP20 (4), which results in an FRT site in place of the antibiotic resistance cassette. Used for time-course assays. |
| Δ*cho* Δ*recA*::*kanR* | MG1655 Δ*cho*::FRT Δ*recA*::*kanR* | This work | Generated through P1 transduction using Δ*cho*::*kanR* from the Keio collection (3) as donor into MG1655 recipient, followed by curing of *kanR* by pCP20 (4), which results in an FRT site in place of the antibiotic resistance cassette. A second round of P1 transduction was conducted using Δ*recA*::*kanR* as donor into Δ*cho*::FRT as recipient. Used for recovery screens. |
| Δ*cho* Δ*uvrC* Δ*recA*::*kanR* | MG1655 Δ*cho*::FRT Δ*uvrC::FRT* Δ*recA::kanR* | This work | Generated through P1 transduction using Δ*cho*::*kanR* from the Keio collection (3) as donor into MG1655 recipient, followed by curing of *kanR* by pCP20 (4), which results in an FRT site in place of the antibiotic resistance cassette. A second round of P1 transduction was conducted using Δ*uvrC*::*kanR* from the Keio collection (3) as donor into Δ*cho*::FRT as recipient, followed by curing of *kanR* by pCP20 (4), which results in an FRT site in place of the antibiotic resistance cassette. A third round of P1 transduction was conducted using Δ*recA*::*kanR* as donor into Δ*cho*::FRT Δ*uvrC*::FRT as recipient. Used for recovery screens. |
| Δ*mutS* Δ*recA*::*kanR* | MG1655 Δ*mutS*::FRT Δ*recA::kanR* | This work | Generated through P1 transduction using Δ*mutS*::*kanR* from the Keio collection (3) as donor into MG1655 recipient, followed by curing of *kanR* by pCP20 (4), which results in an FRT site in place of the antibiotic resistance cassette. A second round of P1 transduction was conducted using Δ*recA*::*kanR* as donor into Δ*mutS*::FRT as recipient. Used for recovery screens. |
| Δ*mutH* Δ*recA*::*kanR* | MG1655 Δ*mutH*::FRT Δ*recA::kanR* | This work | Generated through P1 transduction using Δ*mutH*::*kanR* from the Keio collection (3) as donor into MG1655 recipient, followed by curing of *kanR* by pCP20 (4), which results in an FRT site in place of the antibiotic resistance cassette. A second round of P1 transduction was conducted using Δ*recA*::*kanR* as donor into Δ*mutH*::FRT as recipient. Used for recovery screens. |
| Δ*mutL* Δ*recA*::*kanR* | MG1655 Δ*mutL*::FRT Δ*recA::kanR* | This work | Generated through P1 transduction using Δ*mutL*::*kanR* from the Keio collection (3) as donor into MG1655 recipient, followed by curing of *kanR* by pCP20 (4), which results in an FRT site in place of the antibiotic resistance cassette. A second round of P1 transduction was conducted using Δ*recA*::*kanR* as donor into Δ*mutL*::FRT as recipient. Used for recovery screens. |
| Δ*recB*::*kanR* | MG1655 Δ*recB*::*kanR* | (5) | Generated through P1 transduction using Δ*recB*::*kanR* from the Keio collection (3) as donor into MG1655 recipient. Used for recovery screens. |
| Δ*recB* | MG1655 Δ*recB*::FRT | (5) | Generated by curing Δ*recB*::*kanR* of *kanR* by pCP20 (4), which results in an FRT site in place of the antibiotic resistance cassette. Used for time-course assays. |
| Δ*recB* Δ*uvrD*::*kanR* | MG1655 Δ*recB*::*kanR* ΔuvrD::FRT | This work | Generated through P1 transduction using Δ*recB*::*kanR* as donor into Δ*uvrD*::FRT recipient. Used for recovery screens. |
| Δ*recB* Δ*uvrD* | MG1655 Δ*recB*::FRT Δ*uvrD*::FRT | This work | Generated by curing Δ*uvrD*::FRT Δ*recB::kanR* of *kanR* by pGL04 (6), which results in an FRT site in place of the antibiotic resistance cassette. Used for time-course assays. |
| Δ*recC*::*kanR* | MG1655 Δ*recC*::*kanR* | This work | Generated through P1 transduction using Δ*recC*::*kanR* from the Keio collection (3) as donor into MG1655. Used for recovery screens. |
| Δ*recC* | MG1655 Δ*recC*::FRT | This work | Generated by curing Δ*recC*::*kanR* of *kanR* by pGL04 (6), which results in an FRT site in place of the antibiotic resistance cassette. Used for time-course assays. |
| Δ*recC* Δ*uvrD*::*kanR* | MG1655 Δ*recC*::*kanR* Δ*uvrD*::FRT | This work | Generated through P1 transduction using Δ*recC*::*kanR* as donor into Δ*uvrD*::FRT recipient. Used for recovery screens. |
| Δ*recC* Δ*uvrD* | MG1655 Δ*recC*::*kanR* Δ*uvrD*::FRT | This work | Generated by curing Δ*uvrD*::FRT Δ*recC*::*kanR* of *kanR* by pGL04 (6), which results in an FRT site in place of the antibiotic resistance cassette. Used for time-course assays. |
| Δ*recD* Δ*uvrD*::*kanR* | MG1655 Δ*recD*::FRT Δ*uvrD*::*kanR* | This work | Generated through P1 transduction using Δ*recD*::*kanR* from the Keio collection (3) as donor into MG1655 recipient, followed by curing of *kanR* by pCP20 (4), which results in an FRT site in place of the antibiotic resistance cassette. A second round of P1 transduction was conducted using Δ*uvrD*::*kanR* as donor into Δ*recD*::FRT recipient. Used for recovery screens. |
| Δ*recJ* Δ*uvrD*::*kanR* | MG1655 Δ*recJ*::FRT Δ*uvrD*::*kanR* | This work | Generated through P1 transduction using Δ*recJ*::*kanR* from the Keio collection (3) as donor into MG1655 recipient, followed by curing of *kanR* by pCP20 (4), which results in an FRT site in place of the antibiotic resistance cassette. A second round of P1 transduction was conducted using Δ*uvrD*::*kanR* as donor into Δ*recJ*::FRT recipient. Used for recovery screens. |
| Δ*recN* Δ*uvrD*::*kanR* | MG1655 Δ*recN*::FRT Δ*uvrD*::*kanR* | This work | Generated through P1 transduction using Δ*uvrD*::*kanR* as donor into Δ*recN*::FRT (5) recipient. Used for recovery screens. |
| Δ*recF* Δ*uvrD*::*kanR* | MG1655 Δ*recF*::FRT Δ*uvrD*::*kanR* | This work | Generated through P1 transduction using Δ*uvrD*::*kanR* as donor into Δ*recF*::FRT (5) recipient. Used for recovery screens. |
| Δ*recO* Δ*uvrD*::*kanR* | MG1655 Δ*recO*::FRT Δ*uvrD*::*kanR* | This work | Generated through P1 transduction using Δ*recO*::*kanR* from the Keio collection (3) as donor into MG1655 recipient, followed by curing of *kanR* by pGL04 (6), which results in an FRT site in place of the antibiotic resistance cassette. A second round of P1 transduction was conducted using Δ*uvrD*::*kanR* as donor into Δ*recO*::FRT recipient. Used for recovery screens. |
| Δ*recR* Δ*uvrD*::*kanR* | MG1655 Δ*recR*::FRT Δ*uvrD*::*kanR* | This work | Generated through P1 transduction using Δ*recR::kanR* from the Keio collection (3) as donor into MG1655 recipient, followed by curing of *kanR* by pGL04 (6), which results in an FRT site in place of the antibiotic resistance cassette. A second round of P1 transduction was conducted using Δ*uvrD*::*kanR* as donor into Δ*recR*::FRT recipient. Used for recovery screens. |
| *lexA3* Δ*uvrD* | MG1655 *lexA3* Δ*uvrD::kanR* | *lexA3* mutation was obtained from (7),  double mutant was constructed in this work | Generated through P1 transduction using Δ*uvrD*::*kanR* as donor into *lexA3* (5) recipient. Used for recovery screens. |
| Δ*uvrA* Δ*recB*::*kanR* | MG1655 Δ*uvrA*::FRT Δ*recB*::*kanR* | This work | Generated through P1 transduction using Δ*recB*::*kanR* as donor into Δ*uvrA*::FRT as recipient. Used for recovery screens. |
| Δ*uvrB* Δ*recB*::*kanR* | MG1655 Δ*uvrB*::FRT Δ*recB*::*kanR* | This work | Generated through P1 transduction using Δ*recB*::*kanR* as donor into Δ*uvrB*::FRT as recipient. Used for recovery screens. |
| Δ*mfd* Δ*recB*::*kanR* | MG1655 Δ*mfd*::FRT Δ*recB*::*kanR* | This work | Generated through P1 transduction using Δ*recB*::*kanR* as donor into Δ*mfd*::FRT as recipient. Used for recovery screens. |
| Δ*uvrA* Δ*recC*::*kanR* | MG1655 Δ*uvrA*::FRT Δ*recC*::*kanR* | This work | Generated through P1 transduction using Δ*recC*::*kanR* as donor into Δ*uvrA*::FRT as recipient. Used for recovery screens. |
| Δ*uvrB* Δ*recC*::*kanR* | MG1655 Δ*uvrB*::FRT Δ*recC*::*kanR* | This work | Generated through P1 transduction using Δ*recC*::*kanR* as donor into Δ*uvrB*::FRT as recipient. Used for recovery screens. |
| Δ*mfd* Δ*recC*::*kanR* | MG1655 Δ*mfd*::FRT Δ*recC*::*kanR* | This work | Generated through P1 transduction using Δ*recC*::*kanR* as donor into Δ*mfd*::FRT as recipient. Used for recovery screens. |
| Δ*recF* Δ*uvrA* Δ*recB*::*kanR* | MG1655 Δ*recF*::FRT Δ*uvrA*::FRT Δ*recB*::*kanR* | This work | Generated through P1 transduction using Δ*uvrA*::*kanR* as donor into Δ*recF*::FRT recipient (5), followed by curing of *kanR* by pGL04 (6), which results in an FRT site in place of the antibiotic resistance cassette. A second round of P1 transduction was conducted using Δ*recB*::*kanR* as donor into Δ*recF*::FRT Δ*uvrA*::FRT recipient. Used for recovery screens. |
| Δ*recO* Δ*uvrA* Δ*recB*::*kanR* | MG1655 Δ*recO*::FRT Δ*uvrA*:FRT Δ*recB*::*kanR* | This work | Generated through P1 transduction using Δ*recO::kanR* from the Keio collection (3) as donor into Δ*uvrA*::FRT recipient, followed by curing of *kanR* by pGL04 (6), which results in an FRT site in place of the antibiotic resistance cassette. A second round of P1 transduction was conducted using Δ*recB*:*:kanR* as donor into Δ*uvrA*::FRT Δ*recO*::FRT recipient. Used for recovery screens. |
| Δ*recR* Δ*uvrA* Δ*recB*::*kanR* | MG1655 Δ*recR*::FRT Δ*uvrA*::FRT Δ*recB*::*kanR* | This work | Generated through P1 transduction using Δ*recR::kanR* from the Keio collection (3) as donor into Δ*uvrA*::FRT recipient, followed by curing of *kanR* by pGL04 (6), which results in an FRT site in place of the antibiotic resistance cassette. A second round of P1 transduction was conducted using Δ*recB*:*:kanR* as donor into Δ*uvrA*::FRT Δ*recR*::FRT recipient. Used for recovery screens. |
| Δ*recB* Δ*uvrD* *attTn7*::MCS | MG155 Δ*recB*::FRT Δ*uvrD*::FRT *attTn7*::MCS | This work | Generated by inserting MCS using the Tn7 transposition machinery expressed from pGRG25 into Δ*recB*::FRT Δ*uvrD*::FRT, followed by curing of temperature sensitive plasmid (8). Used for complementation assays. |
| Δ*recB* Δ*uvrD* *attTn7*::P_uvrD_-*uvrD* | MG1655 Δ*recB*::FRT Δ*uvrD*::FRT *attTn7*::P_uvrD_-*uvrD* | This work | Generated by inserting P_uvrD_-*uvrD* using the Tn7 transposition machinery expressed from pGRG25 P_uvrD_-*uvrD* into Δ*recB*::FRT Δ*uvrD*::FRT, followed by curing of temperature sensitive plasmid (8). Used for complementation assays. |
| Δ*recC* Δ*uvrD* *attTn7*::MCS | MG1655 Δ*recC*::FRT Δ*uvrD*::FRT *attTn7*::MCS | This work | Generated by inserting MCS using the Tn7 transposition machinery expressed from pGRG25 into Δ*recC*::FRT Δ*uvrD*::FRT, followed by curing of temperature sensitive plasmid (8). Used for complementation assays. |
| Δ*recC* Δ*uvrD* *attTn7*::P_uvrD_-*uvrD* | MG1655 Δ*recC*::FRT Δ*uvrD*::FRT *attTn7*::P_uvrD_-*uvrD* | This work | Generated by inserting P_uvrD_-*uvrD* using the Tn7 transposition machinery expressed from pGRG25 P_uvrD_-*uvrD* into Δ*recC*::FRT Δ*uvrD*::FRT, followed by curing of temperature sensitive plasmid (8). Used for complementation assays. |

**References:**

1.	Kohanski MA, Dwyer DJ, Hayete B, Lawrence CA, Collins JJ. A Common Mechanism of Cellular Death Induced by Bactericidal Antibiotics. Cell. 2007;130(5):797-810.

2.	Lemma AS, Brynildsen MP. Toxin Induction or Inhibition of Transcription or Translation Posttreatment Increases Persistence to Fluoroquinolones. mBio. 2021;12(4).

3.	Baba T, Ara T, Hasegawa M, Takai Y, Okumura Y, Baba M, et al. Construction of Escherichia coli K-12 in-frame, single-gene knockout mutants: the Keio collection. Molecular Systems Biology. 2006;2:2006.0008.

4.	Cherepanov PP, Wackernagel W. Gene disruption in Escherichia coli: TcR and KmR cassettes with the option of Flp-catalyzed excision of the antibiotic-resistance determinant. Gene. 1995;158(1):9-14.

5.	Lemma AS, Soto-Echevarria N, Brynildsen MP. Fluoroquinolone Persistence in Escherichia coli Requires DNA Repair despite Differing between Starving Populations. Microorganisms. 2022;10(2):286.

6.	Leon G, Brynildsen MP. Conservation and divergence of ciprofloxacin persister survival mechanisms between Pseudomonas aeruginosa and Escherichia coli. PLOS Genetics. 2025;21(9):e1011840.

7.	Völzing KG, Brynildsen MP. Stationary-Phase Persisters to Ofloxacin Sustain DNA Damage and Require Repair Systems Only during Recovery. mBio. 2015;6(5):e00731-15.

8.	McKenzie GJ, Craig NL. Fast, easy and efficient: site-specific insertion of transgenes into Enterobacterial chromosomes using Tn7 without need for selection of the insertion event. BMC Microbiology. 2006;6(1):39.
