# Supplementary material for: Genetic analysis of epistasis between nucleotide excision repair and homologous recombination in the recovery of persisters after fluoroquinolone treatment
Source: PLoS Genet. 2026 Sep 21;22(9):e1012305. doi: 10.1371/journal.pgen.1012305 (PMC13592689; doi:10.1371/journal.pgen.1012305)
Supplement: Supplementary file 2 [file pgen.1012305.s006.docx]

**Supplemental table 2:** List of primers (5’ 🡪 3’) used to verify genetic modifications.

| **Gene** | **Internal forward primer** | **Internal reverse primer** | **External forward primer** | **External reverse primer** | **Source(s)** |
| --- | --- | --- | --- | --- | --- |
| **Δ*recA*** | TGGAAACCATCTCTACCGGTTC | GACGAACAGAGGCGTAGAAT | CTGGTTTGCTTTTGCCACTG | AATACGCGCAGGTCCATAAC | (1) |
| **Δ*uvrD*** | TAATGACAAACAGCGCGAAG | CAGACGCCCGTTATTGTTTT | TTACTGCCGCATCTGGAAAT | TAGGCCAAATAAGGTGCGCA | Primers were sourced from Lemma et al. (2022) (1), with the exception of the external reverse primer which was  generated for this work. |
| **Δ*uvrA*** | AAAGGCGAACACACCAAAAC | AATTCCATCGCATGACCAAT | CACAACACTCCGGGTAATGC | ACCGTGTATCTCGACGGTTC | This work |
| **Δ*uvrB*** | GTCTGGGCGATCCTGATTTA | CGTAATTCCGTGTTCCTCGT | CGGTAAGGCCGGAGTTTTAT | GACAAAGTGCGCGTTGTTTA | This work |
| **Δ*uvrC*** | CGCGTTACAACGTTTTGCTA | GCTCAAAGAACAGCGTTTCC | AACATTCATGGCGATGTTGA | CCAGGTGACAGGCAGATAAAA | This work |
| **Δ*mfd*** | CCGTGAGTATGACAGCATGG | AGCTTCCTGATCCCCAGTTT | GCATTCAGCGATCTGTTGTT | CGTTAGCCAGCGAGAAAAAC | This work |
| **Δ*cho*** | CGCTCTATATCGGCAAAAGC | CCTCATGGCTCTCTTTACCG | GTGAACGCCTTATTCGACCT | AAGTCAGAATTGCCGAACGTA | This work |
| **Δ*mutS*** | CCGGATGGGTGATTTTTATG | CCGGATCAAGATTTTCCAGA | TAAGCATCACGCAAAAGCTG | AGCGTGTAGATGGCATGGTT | This work |
| **Δ*mutH*** | TCTCTCCTCCCGAAACTGAA | AGAAAATGACGGGCCAGTAG | GAATTGAACAACGCATGTGG | GATCCCAAGAACGATCTCCA | This work |
| **Δ*mutL*** | AGGTCTTACCGCCACAACTG | AGTTGCGGACATAACCGTTC | CAGCAACAACAGCGAAGAAC | GTCCCGATATCCATCCCTTT | This work |
| **Δ*recB*** | GCGGAAGATCTGCGTTTGCT | TCATAGCGGTGTGCCTGCAT | GAAGGCTGGAAAGTGTGGGA | GCGACGCCTCGTTATTTTCC | Internal reverse primer was sourced from Lemma et. al (2022) (1), remaining primers were generated for this work. |
| **Δ*recC*** | ACCGCGCCAATCTCTATCAG | CAGCCCTTCTTCGGTGATGT | CCAGTACCAACAGCTCTGGC | TCCCGTTTCTGCCTGACTTAA | This work |
| **Δ*recD*** | AATCGCATGTGGTGTAACGA | ATCAAGATGCAGCGGGTTAC | CGTGGCGTTGATAAAGAACA | TCCAGGAACGTGGATTTACC | This work |
| **Δ*recJ*** | AACTGAGCGGCGTCGAAAAG | TGATGCGCGAAGCCAGAATA | CCAGTTGCGACGTGGATATT | TTCCAGACGCTCTTTCTTGG | (2) |
| **Δ*recN*** | TGTTTGCTTCGTCGCGTGAT | CATTTTACGCGCCGTGATGA | CACCAAGCTCGGCTGGTCAA | GCAGCAGTCAGCGTTTTACA | (1) |
| **Δ*recF*** | TCCCTCACCCGCTTGTTGAT | TGATCGCGCTGACAAAGACC | GCGAAAACGTCCGCATGATG | TATACATACCCGGGCGCTTA | (1) |
| **Δ*recO*** | TCACCCCTCTCTTGCTACGT | TGTTTTCACCGTTCGCTTAGG | GACCGTGGAGATCGAACGTT | CACCGCCATCTCCAGATTCA | This work |
| **Δ*recR*** | CAAACCAGCCCGCTGTTAAC | AATCTTATGACGCCCGGCAA | CGCAGCACGTCGTATTGAAG | GGATGTGGGAGAGATGGGGA | This work |
| ***attTn7*** | N/A | N/A | GATGACGGTTTGTCACATGGA | GATGCTGGTGGCGAAGCTGT | (3) |
| ***kanR*** | N/A | ATGATGGATACTTTCTCGGCAGGAG | N/A | N/A | (1) |

**References:**

1.	Lemma AS, Soto-Echevarria N, Brynildsen MP. Fluoroquinolone Persistence in Escherichia coli Requires DNA Repair despite Differing between Starving Populations. Microorganisms. 2022;10(2):286.

2.	Tang J, Herzfeld AM, Leon G, Brynildsen MP. Differential impacts of DNA repair machinery on fluoroquinolone persisters with different chromosome abundances. mBio.15(5):e00374-24.

3.	McKenzie GJ, Craig NL. Fast, easy and efficient: site-specific insertion of transgenes into Enterobacterial chromosomes using Tn7 without need for selection of the insertion event. BMC Microbiology. 2006;6(1):39.
