# Supplementary material for: Genetic analysis of epistasis between nucleotide excision repair and homologous recombination in the recovery of persisters after fluoroquinolone treatment
Source: PLoS Genet. 2026 Sep 21;22(9):e1012305. doi: 10.1371/journal.pgen.1012305 (PMC13592689; doi:10.1371/journal.pgen.1012305)
Supplement: Supplementary file 3 [file pgen.1012305.s007.docx]

**Supplementary Table 3:** List of plasmids used in this study.

| **Plasmid** | **Relevant genotype** | **Source** | **Notes** |
| --- | --- | --- | --- |
| **pCP20** | *ampR* and *camR*, temperature sensitive replication (repA101ts), *flp* | (1) | Cloning plasmid for curing FRT-flanked resistance markers. |
| **pGL04** | Contains RSF1010 oriV, *ampR*, *flp,* *sacB* | (2) | Cloning plasmid for curing FRT-flanked resistance markers. |
| **pUA66** | pSC101 ori, *kanR*, promoterless *gfpmut2* | (3) | Empty vector |
| **pUA66-P_recA_-*recA*** | pUA66 P_recA_-*recA, kanR* | (4) | Complementation plasmid. Insertion of P_recA_-*recA* replaced *gfpmut2* in pUA66. |
| **pUA66-P_uvrA_-*uvrA*** | pUA66 P_uvrA_-*uvrA, kanR* | This work | Complementation plasmid. Insertion of P_uvrA_-*uvrA* replaced *gfpmut2* in pUA66. |
| **pUA66-P_uvrB_-*uvrB*** | pUA66 P_uvrB_-*uvrB, kanR* | This work | Complementation plasmid. Insertion of P_uvrB_-*uvrB* replaced *gfpmut2* in pUA66. |
| **pUA66-P_mfd_-*mfd*** | pUA66 P_mfd_-*mfd, kanR* | This work | Complementation plasmid. Insertion of P_mfd_-*mfd* replaced *gfpmut2* in pUA66. |
| **pUA66-P_uvrD_-*uvrD*** | pUA66 P_uvrD_-*uvrD, kanR* | (5) | Complementation plasmid. Insertion of P_uvrD_-*uvrD* replaced *gfpmut2* in pUA66. |
| **pUA66-P_recB_-*recB*** | pUA66 P_recB_-*recB, kanR* | (4) | Complementation plasmid. Insertion of P_recB_-*recB* replaced *gfpmut2* in pUA66. |
| **pUA66-P_recC_-*recC*** | pUA66 P_recC_-*recC, kanR* | This work | Complementation plasmid. Insertion of P_recC_-*recC* replaced *gfpmut2* in pUA66. |
| **pGRG25** | *ampR*, temperature sensitive replication (repA101ts), Tn7 transposition machinery | (6) | Plasmid for inserting transpositions at the *attTn7* site. |
| **pGRG25-P_uvrD_-*uvrD*** | pGRG25 P_uvrD_-*uvrD, ampR* | This work | Complementation plasmid. P_uvrD_-*uvrD* was inserted into the MCS of pGRG25. |
| **pUA66-P_recA_-*recA*(G204S)** | pUA66-P_recA_-*recA*(G204S), *kanR* | This work | Complementation plasmid.  The G204S mutation was inserted into the *recA* gene of pUA66 P_recA_-*recA* via site directed mutagenesis (SDM). |
| **pUA66-P_recA_-*recA*(N304D)** | pUA66-P_recA_-*recA*(N304D), *kanR* | This work | Complementation plasmid.  The N304S mutation was inserted into the *recA* gene of pUA66 P_recA_-*recA* via SDM. |
| **pUA66-P_uvrD_-*uvrD*(ΔCTD)** | pUA66-P_uvrD_-*uvrD***(**ΔCTD) | This work | Complementation plasmid. The CTD was deleted from the *uvrD* gene of pUA66 P_uvrD_-*uvrD* via SDM. This corresponded to removal of amino acid residues 645-720 |
| **pUA66-P_uvrD_-*uvrD*(R396E)** | pUA66-P_uvrD_-*uvrD*(R396E) | This work | Complementation plasmid.  The R396E mutation was inserted into the *uvrD* gene of pUA66 P_uvrD_-*uvrD* via SDM. |
| **pUA66-P_uvrD_-*uvrD*(R284A)** | pUA66-P_uvrD_-*uvrD*(R284A) | This work | Complementation plasmid.  The R284a mutation was inserted into the *uvrD* gene of pUA66 P_uvrD_-*uvrD* via SDM. |

**References:**

1.	Cherepanov PP, Wackernagel W. Gene disruption in Escherichia coli: TcR and KmR cassettes with the option of Flp-catalyzed excision of the antibiotic-resistance determinant. Gene. 1995;158(1):9-14.

2.	Leon G, Brynildsen MP. Conservation and divergence of ciprofloxacin persister survival mechanisms between Pseudomonas aeruginosa and Escherichia coli. PLOS Genetics. 2025;21(9):e1011840.

3.	Zaslaver A, Bren A, Ronen M, Itzkovitz S, Kikoin I, Shavit S, et al. A comprehensive library of fluorescent transcriptional reporters for Escherichia coli. Nature Methods. 2006;3(8):623-8.

4.	Mok WWK, Brynildsen MP. Timing of DNA damage responses impacts persistence to fluoroquinolones. Proceedings of the National Academy of Sciences. 2018;115(27).

5.	Tang J, Herzfeld AM, Leon G, Brynildsen MP. Differential impacts of DNA repair machinery on fluoroquinolone persisters with different chromosome abundances. mBio.15(5):e00374-24.

6.	McKenzie GJ, Craig NL. Fast, easy and efficient: site-specific insertion of transgenes into Enterobacterial chromosomes using Tn7 without need for selection of the insertion event. BMC Microbiology. 2006;6(1):39.
