# Supplementary material for: Genetic analysis of epistasis between nucleotide excision repair and homologous recombination in the recovery of persisters after fluoroquinolone treatment
Source: PLoS Genet. 2026 Sep 21;22(9):e1012305. doi: 10.1371/journal.pgen.1012305 (PMC13592689; doi:10.1371/journal.pgen.1012305)
Supplement: Supplementary file 4 [file pgen.1012305.s008.docx]

**Supplementary Table 4:** List of primers used for plasmid construction.

| **Primer name** | **Forward primer** | **Reverse primer** | **Description** | **Source(s)** |
| --- | --- | --- | --- | --- |
| pUA66 backbone | CCTGCAGGCATGCAAGCTC | CTCGAGGTGAAGACGAAAGGG | Used to amplify pUA66 backbone to generate plasmid derivatives with Gibson assembly (1). | (2) |
| pUA66 P_uvrA_-*uvrA* | CCTTTCGTCTTCACCTCGAGTGTTCGTGTCTCCTGAAAAAAATCGTTCT | AGAGCTTGCATGCCTGCAGGTTACAGCATCGGCTTAAGGAAGCGTGC | Used to amplify P_uvrA_-*uvrA* from the MG1655 genome for integration into pUA66 with Gibson assembly (1). | This work |
| pUA66 P_uvrB_-*uvrB* | CCTTTCGTCTTCACCTCGAGTTATCCACTATTCCTGTGGATAACCATGTG | AGAGCTTGCATGCCTGCAGGTTACGATGCCGCGATAAACAGCTCACGCAGC | Used to amplify P_uvrB_-*uvrB* from the MG1655 genome for integration into pUA66 with Gibson assembly (1). | This work |
| pUA66 P_mfd_-*mfd* | CCTTTCGTCTTCACCTCGAGAGCCGGGGCGATTCAATTTGCTAAACCATG | AGAGCTTGCATGCCTGCAGGTTAAGCGATCGCGTTCTCTTCCAGTTCACGCAT | Used to amplify P_mfd_-*mfd* from the MG1655 genome for integration into pUA66 with Gibson assembly (1). | This work |
| pUA66 P_recC_-*recC* | CCTTTCGTCTTCACCTCGAGTCAGCTTCCCTGAAGAATCAACAAGG | AGAGCTTGCATGCCTGCAGGTCATGACTGATTAAAGCGAAACAGCGG | Used to amplify P_recC_-*recC* from the MG1655 genome for integration into pUA66 with Gibson assembly (1). | This work |
| pGRG25 backbone fragment 1 | AGATTAAATCAGAACGCAGAAGCG | GTGGCTTACTAGGATCCGATATCA | Used to amplify fragment 1 of the pGRG25 backbone to generate pGRG25 P_uvrD_-*uvrD* with Gibson assembly (1). | (3) |
| pGRG25 backbone fragment 2 | TCTCTCATTTTGGGAGGTTTGG | TCTGCGTTCTGATTTAATCTGTATCA | Used to amplify fragment 2 of the pGRG25 backbone to generate pGRG25 P_uvrD_-*uvrD* via Gibson assembly (1). | (3) |
| pGRG25 backbone fragment 3 | GCCACTCGAGCACCTAG | AACCTCCCAAAATGAGAGACC | Used to amplify fragment 3 of the pGRG25 backbone to generate pGRG25 P_uvrD_-*uvrD* via Gibson assembly (1). | (3) |
| pGRG25 P_uvrD_-*uvrD* | GTGGCGCGCCTCCTAGGTGCCGTTTTTACCGGCCTTTGGG | TAATCAGATCCCTCAATAGCCGCATCAGCTACTCATTGGT | Used to amplify P_uvrD_-*uvrD* from the MG1655 genome to generate pGRG25 P_uvrD_-*uvrD* via Gibson assembly (1). | This work |
| pUA66 P_recA_-*recA*(G204S) | TGTGATGTTCagtAACCCGGAAAC | CCAATTTTCATACGGATCTGGTTG | Used to amplify pUA66 P_recA_-*recA* to generate pUA66 P_recA_-*recA*(G204S) via SDM. | This work |
| pUA66 P_recA_-*recA*(N304D) | AGGGTAAAGCGgatGCGACTG | TGACCGATCTTCTCACCTTTGTAGC | Used to amplify pUA66 P_recA_-*recA* to generate pUA66-P_recA_-*recA* N304D via SDM. | This work |
| pUA66 P_uvrD_-*uvrD*(ΔCTD) | TAACGTTGCCGGATGCGGTGCT | CACCTCTTCCACACACT | Used to amplify pUA66 P_uvrD_-*uvrD* to generate pUA66 P_uvrD_-*uvrD* ΔCTD via SDM. | This work |
| pUA66 P_uvrD_-*uvrD*(R396E) | GCTCTCGTATCTGgaaCTGATTGCCAACCGCAA | GCATCTTTGATTTCCTGGCG | Used to amplify pUA66 P_uvrD_-*uvrD* to generate pUA66 P_uvrD_-*uvrD* R396E via SDM. | This work |
| pUA66 P_uvrD_-*uvrD*(R284A) | GCAAAACTACgcgTCTACCAGCAATATTC | TCCAGACGAATAGTTTCGGC | Used to amplify pUA66 P_uvrD_-*uvrD* to generate pUA66 P_uvrD_-*uvrD* R284E via SDM. | This work |

**References:**

1.	Gibson DG, Young L, Chuang R-Y, Venter JC, Hutchison III CA, Smith HO. Enzymatic assembly of DNA molecules up to several hundred kilobases. Nature Methods. 2009;6:343–5.

2.	Tang J, Herzfeld AM, Leon G, Brynildsen MP. Differential impacts of DNA repair machinery on fluoroquinolone persisters with different chromosome abundances. mBio.15(5):e00374-24.

3.	Sniezek KG, Brynildsen MP. An investigation of gene dosage reveals that increased sensitivity to D-cycloserine divergently impacts the transience of heteroresistance in Escherichia coli.
